# Identifying aphid resistance in the ancestral wheat *Triticum monococcum* under field conditions

**DOI:** 10.1038/s41598-021-92883-9

**Published:** 2021-06-29

**Authors:** Amma L. Simon, John C. Caulfield, Kim E. Hammond-Kosack, Linda M. Field, Gudbjorg I. Aradottir

**Affiliations:** 1 Department of Biointeractions and Crop Protection, Rothamsted Research, Harpenden, AL5 2JQ Hertfordshire UK; 2 Division of Plant and Crop Sciences, School of Biosciences, University of Nottingham, Loughborough, LE12 5RD Leicestershire UK; 3 Department of Pathology, NIAB, Lawrence Weaver Road, Cambridge, CB3 0LE UK

**Keywords:** Ecology, Agroecology, Entomology

## Abstract

Wheat is an economically, socially, and nutritionally important crop, however, aphid infestation can often reduce wheat yield through feeding and virus transmission. Through field phenotyping, we investigated aphid resistance in ancestral wheat *Triticum monococcum* (L.). Aphid (*Rhopalosiphum padi* (L.)*, Sitobion avenae* (F.) and *Metopolophium dirhodum* (Wlk.)) populations and natural enemy presence (parasitised mummified aphids, ladybird adults and larvae and lacewing eggs and larvae) on two naturally susceptible wheat varieties, *Triticum aestivum* (L.) var. Solstice and *T. monococcum* MDR037, and three potentially resistant genotypes *T. monococcum* MDR657, MDR045 and MDR049 were monitored across three years of field trials. *Triticum monococcum* MDR045 and MDR049 had smaller aphid populations, whereas MDR657 showed no resistance. Overall, natural enemy presence was positively correlated with aphid populations; however, MDR049 had similar natural enemy presence to MDR037 which is susceptible to aphid infestation. It is hypothesised that alongside reducing aphid population growth, MDR049 also confers indirect resistance by attracting natural enemies. The observed resistance to aphids in MDR045 and MDR049 has strong potential for introgression into commercial wheat varieties, which could have an important role in Integrated Pest Management strategies to reduce aphid populations and virus transmission.

## Introduction

Wheat *Triticum aestivum* (L.) is the third most important crop globally and is the main ingredient in many diets worldwide^1,2^ providing 20–29% of dietary calories and protein^3^. In Europe, wheat is the main crop with 138.3 million tonnes produced in 2018 (FAO, 2020). Wheat is vulnerable to biotic stressors including insect pests^4^ which can reduce yield and affect global wheat prices. With such fluctuations, it is evident that preventing and protecting the effects of pest infestations is of great economic, social, and nutritional importance.

Aphids are insects that belong to the superfamily Aphidoidea which are believed to have evolved 280 million years ago. Most of the ~ 6,000 extant species are small soft-bodied insects that feed by piercing the phloem of plants and taking up the sap^5^. This sap removal directly damages the plants through nutrient removal, aphids also indirectly damage plants by transmitting viruses such as the Barley Yellow Dwarf Virus (BYDV) which negatively affects the plant growth and development. Lastly, aphids secrete sucrose rich honeydew which can attract saprophytic fungi and reduce the photosynthetic ability of the host plant^6^.

In Europe, the most prominent and economically important cereal aphids are the Bird cherry-oat aphid (*Rhopalosiphum padi* (L.)), English grain aphid (*Sitobion avenae* (F.)) and the Rose grain aphid (*Metopolophium dirhodum* (Wlk.)). All three species are known to vector BYDV^7–11^ and can on their own and in combination with virus transmission cause between 5 and 80% yield loss^7,12,13^.

Cereal aphid populations are currently controlled predominantly by insecticides^10^, however, there are increased reports of resistance towards certain classes of insecticides in cereal aphids^14^. These, coupled with restrictions on insecticidal classes that farmers are allowed use, leave fewer options to curb aphid damage to the wheat crop, ultimately affecting food security of wheat^15,16^. Aphid populations are predicted to increase due to climate change; this will have a knock on adverse effect on the agricultural economy through negative effects on yield and quality^17,18^. The combination of these factors affecting wheat food security, has increased the focus on alternative methods of aphid control including the use of wheat with natural resistance as part of integrated pest management (IPM).

Plants have various defence mechanisms against aphid damage. Studies have shown that resistance traits are under selection from herbivores, and both the plant defence theory and plant–herbivore coevolutionary theory assume that resistance traits have evolved as adaptations to reduce herbivory^19–23^.

Resistance to aphids can be direct or indirect, where in the latter resistance is mediated via another organism. Direct resistance can be pre-alighting (antixenosis) where visual or olfactory cues from the plant affect insect behaviour, usually displayed as non-host behavioural responses^24–26^. Another form of direct resistance is post-alighting (antibiosis), aphids feeding on these plants have reduced development and are less fecund. Antixenosis can be used in indirect resistance where visual and/or olfactory cues cause increased numbers of natural enemies, increasing predation and/or parasitism of pest insects^26^.

No commercial varieties have been bred specifically for *R. padi, S. avenae* or *M. dirhodum* resistance to date, but partial resistance has been identified in a number of varieties, including resistance to *R. padi* in Watkins land race collections^27,28^, north-eastern European Gediflux collection^27^ and *S. avenae* resistance in the ancestral diploid *Triticum urartu* (T.) and *Triticum boeoticum* (L.)^29^. The diploid ancestral *Triticum monococcum* (L.) (genome A^m^A^m^)^30,31^ was cultivated in the Neolithic age^32^, genotypes within this species have shown to confer resistance to *R. padi*^33–35^*, S. avenae*^29,31,35–37^ and *M. dirhodum*^30,34^. Antibiosis to both *R. padi* and *S. avenae* has been shown in *T. monococcum* MDR045 and MDR049^33,38^ (Simon et al., unpublished), and antibiosis to *R. padi* in *T. monococcum* MDR657^33^, under laboratory conditions. Aphid susceptible *Triticum aestivum* (L.) Solstice and *T. monococcum* MDR037 were used as controls in these studies. Both antixenosis and antibiosis has been observed towards *M. dirhodum*^30^ and *S. avenae*^31^ in *T. monococcum* under field conditions, however, the genotypes used in these studies were not disclosed^30,31^.

Indirect resistance is mediated via another organism such as a natural enemy. Aphid natural enemies include parasitoids, lacewings, and ladybirds which have been proven to be an effective biocontrol method of aphids in field settings on upland cotton (*Gossypium hirsutum* (L.))^39^ and barley (*Hordeum vulgare* (L.))^40^. In a field study on *T. aestivum*, predator and parasitoid presence resulted in 2.9 to 11.2 times lower Russian wheat aphid (*Diuraphis noxia* (K.)) populations^41^. Ladybirds were one of the most abundant predators observed in this study^41^. It is unknown whether any of the *T. monococcum* genotypes confer indirect resistance to reduce aphid populations through increased presence of natural enemies.

The aim of this study was to determine whether *T. monococcum* MDR657, MDR045 and MDR049 confer direct and/or indirect resistance to economically important aphid species *R. padi*, *S. avenae* and *M. dirhodum* under field conditions. Wheat with natural resistance traits would be desirable candidates for breeding wheat cultivars with resistance to aphids, therefore identifying *T. monococcum* genotypes with such resistance is of great importance.

## Results

### Aphid populations

The aphids investigated were *Rhopalosiphum padi*, *Sitobion avenae* and *Metopolophium dirhodum*, and made up 15%, 62.3% and 22.7% of field trial aphid populations respectively. To identify resistance to these three cereal aphid species, their population densities were investigated.

### Rhopalosipum padi populations

**Figure 1:**
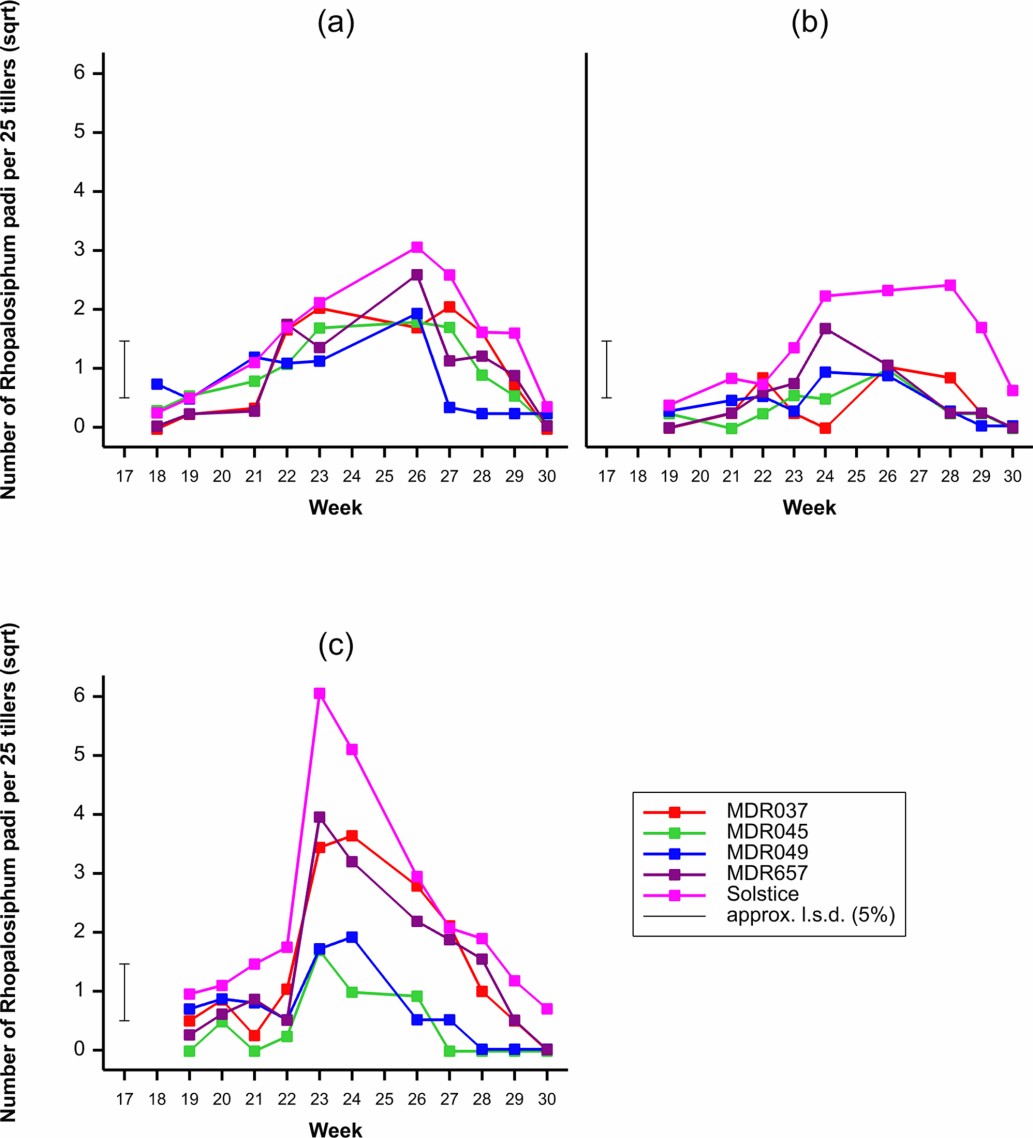
Average number of *Rhopalosiphum padi,* per 25 tillers observed on *Triticum aestivum* Solstice, *Triticum moncoccum* MDR037, MDR657, MDR045 and MDR049 in field trial (**a**) Year 1 (2017), (**b**) Year 2 (2018), (**c**) Year 3 (2019) from week 18 to week 30. All data was subject to square root transformation.

*Rhopalosiphum padi* populations were dependant on the wheat genotype (*F*_4, 599_ = 8.7, *P* < 0.001), with the lowest populations observed on MDR045 and MDR049 (Fig. 1). Populations were also dependant on the field trial year (*F*_2, 599_ = 6.22, *P* < 0.001) and week (*F*_11, 599_ = 11.41, *P* < 0.001) (Fig. 1) with the highest populations observed in third field trial year and week 23 respectively. There was an interaction between the wheat genotype and week (*F*_44, 599_ = 2.02, *P* < 0.001), week and year (*F*_16, 599_ = 3.68, *P* < 0.001) and wheat genotype, week and year (*F*_64, 599_ = 1.36, *P* = 0.042). There was no interaction between the wheat genotype and year (*F*_8, 599_ = 1.12, *P* = 0.353).

### Sitobion avenae populations

**Figure 2:**
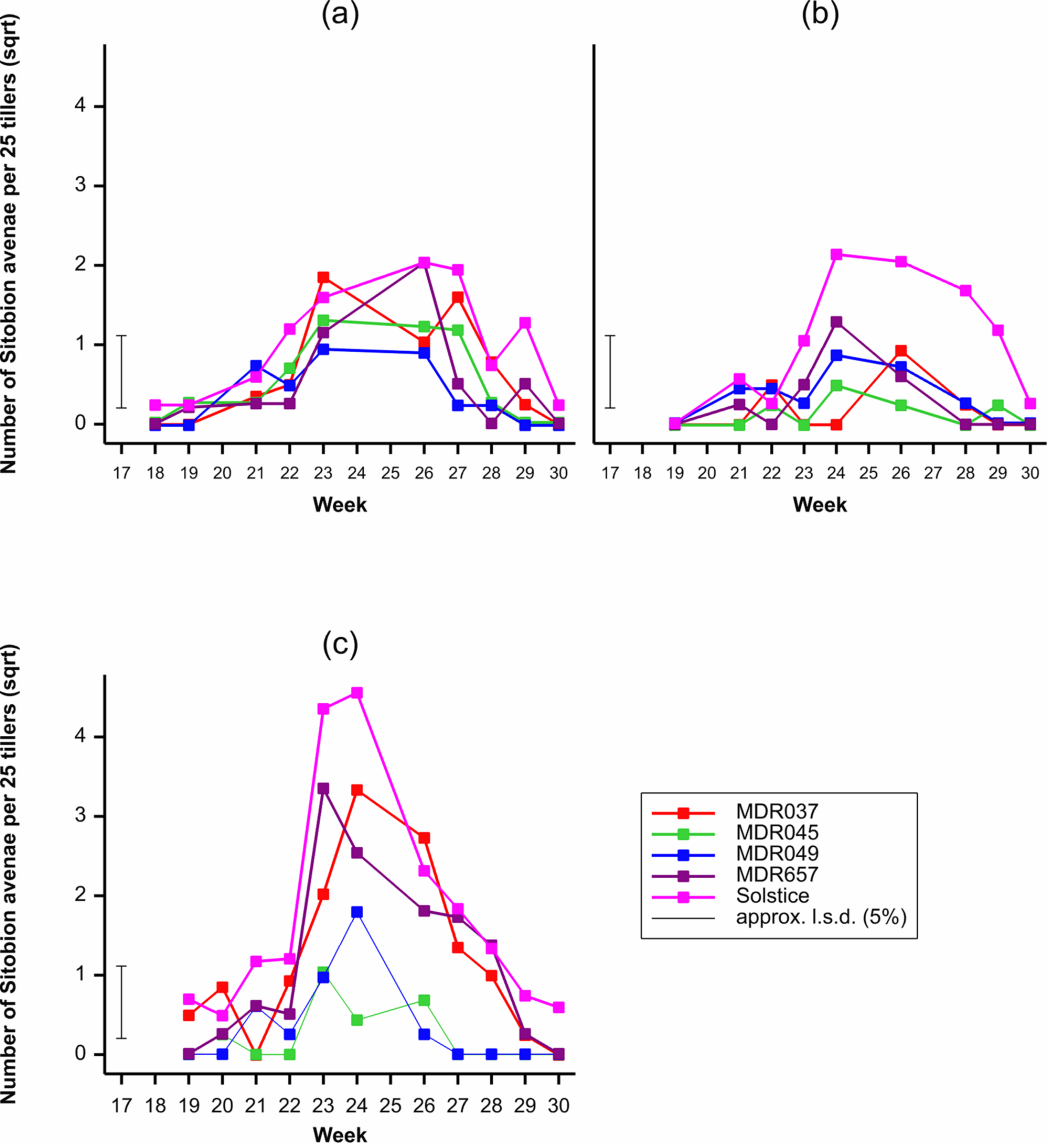
Average number of *Sitobion avenae* per 25 tillers observed on *Triticum aestivum* Solstice, *Triticum moncoccum* MDR037, MDR657, MDR045 and MDR049 in field trial (**a**) Year 1 (2017), (**b**) Year 2 (2018), (**c**) Year 3 (2019) from week 18 to week 30. All data was subject to square root transformation.

*Sitobion avenae* populations were dependant on the wheat genotype (*F*_4, 599_ = 39.4, *P* < 0.001) with the lowest populations observed on MDR045 and MDR049 (Fig. 2). Populations were also dependant on the field trial year (*F*_2, 599_ = 37.42, *P* < 0.001) and week (*F*_11, 599_ = 36.88, *P* < 0.001) (Fig. 2) with the highest populations observed in the third field trial year and week 24 respectively. There was an interaction between the wheat genotype and week (*F*_44, 599_ = 2.6, *P* < 0.001), wheat genotype and year (*F*_8, 599_ = 5.9, *P* < 0.001), week and year (*F*_16, 599_ = 6.48, *P* < 0.001) and wheat genotype, week and year (*F*_64, 599_ = 1.63, *P* = 0.003).

### Metopolophium dirhodum populations

**Figure 3:**
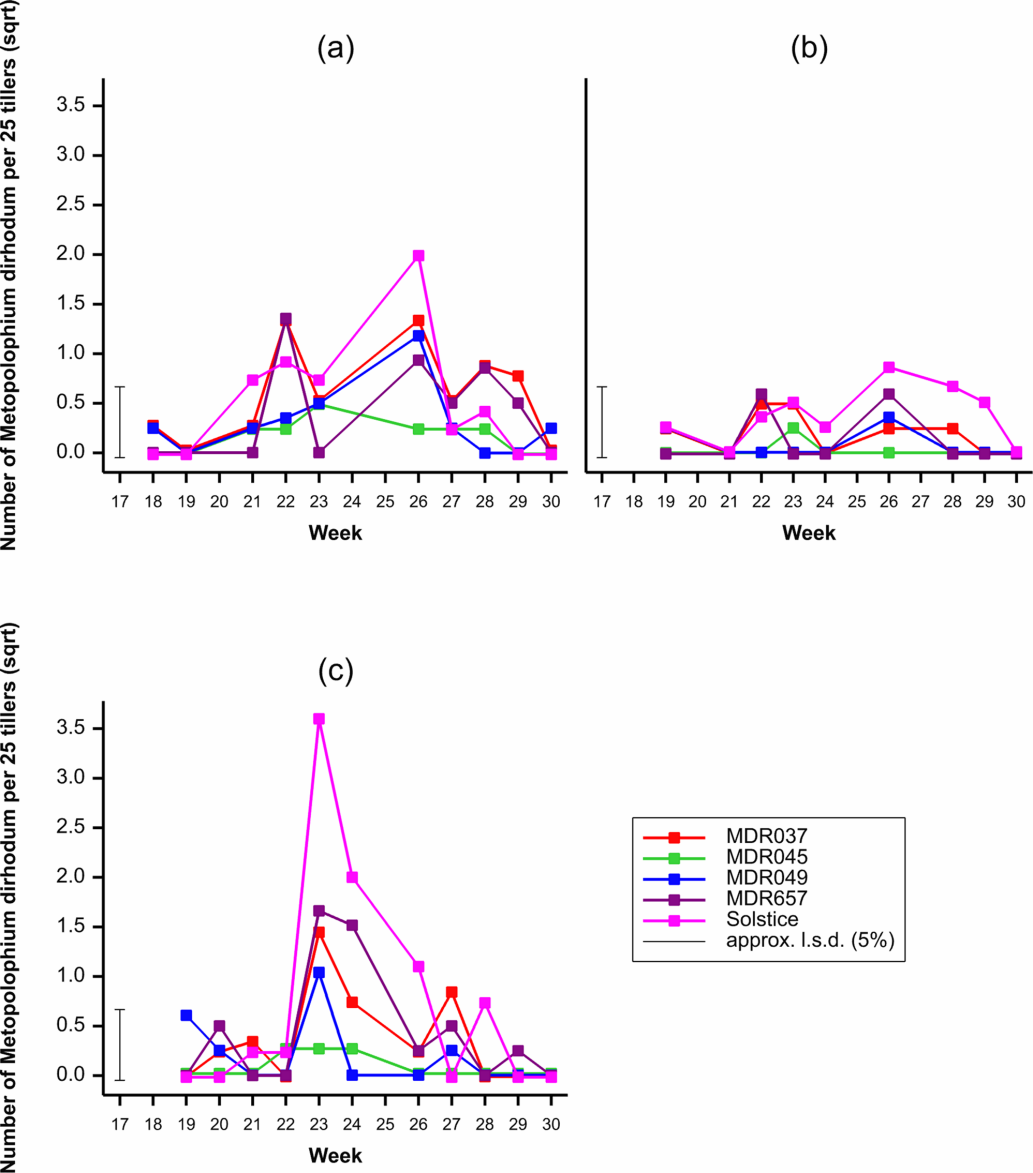
Average number of *Metopolophium dirhodum* per 25 tillers observed on *Triticum aestivum* Solstice, *Triticum moncoccum* MDR037, MDR657, MDR045 and MDR049 in field trial (**a**) Year 1 (2017), (**b**) Year 2 (2018), (**c**) Year 3 (2019) from week 18 to week 30. All data was subject to square root transformation.

*Metopolophium dirhodum* populations were dependant on the wheat genotype (*F*_4, 599_ = 17.16, *P* < 0.001), with the lowest populations observed on MDR045 (Fig. 3). Populations were dependant on the field trial year (*F*_2, 599_ = 16.73, *P* < 0.001) and week (*F*_11, 599_ = 12.18, *P* < 0.001) with the highest populations observed in the third field trial year and week 26 respectively (Fig. 3). There was an interaction between the wheat genotype and week (*F*_44, 599_ = 2.36, *P* < 0.001), week and year (*F*_16, 599_ = 9.58, *P* < 0.001) and wheat genotype, week and year (*F*_64, 599_ = 1.76, *P* < 0.001). There was no interaction between the wheat genotype and year (*F*_8, 599_ = 1.84, *P* = 0.08).

### Alate aphid populations

**Figure 4:**
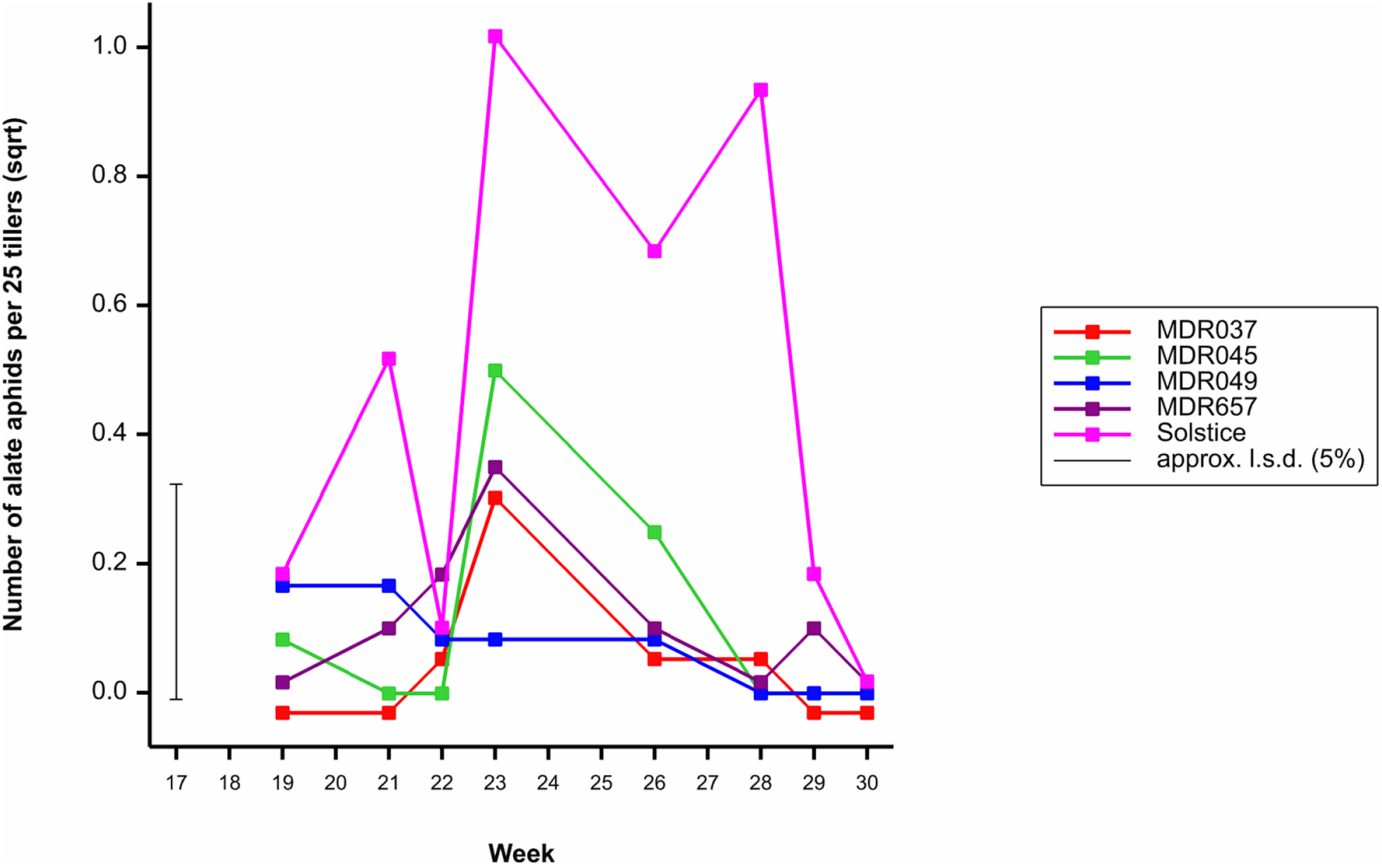
The average number of alate aphids observed on *Triticum aestivum* Solstice, *Triticum moncoccum* MDR037, MDR657, MDR045 and MDR049 from week 18 to week 30 across all three field trials. All data was subject to square root transformation.

Alate (winged) aphids are often the initial colonisers of cereal crops and in our studies made up 6.5% of the field trial aphid populations. To determine the presence of pre-alighting resistance (antixenosis) the alate aphid populations were investigated. Alate aphid density varied depending on the wheat genotype (*F*_4, 599_ = 8.55, *P* < 0.001), the highest density of alate aphids was observed on Solstice (Fig. 4). Alate aphid population density varied by week (*F*_11, 599_ = 4.86, *P* < 0.001), the highest number of alate aphids was observed in week 23 (Fig. 4). There was no difference in alate aphid density between the three years of field trials (*F*_2, 599_ = 1.65, *P* = 0.193). There was no interaction between the wheat genotype and week (*F*_44, 599_ = 1.35, *P* = 0.074), wheat genotype and year (*F*_8, 599_ = 0.25, *P* = 0.982), week and year (*F*_16, 599_ = 1.25, *P* = 0.226), nor wheat genotype, week and year (*F*_64, 599_ = 0.91, *P* = 0.673). Due to low numbers, the average number of alate aphids across the three field trials is presented.

### Natural enemies

**Figure 5:**
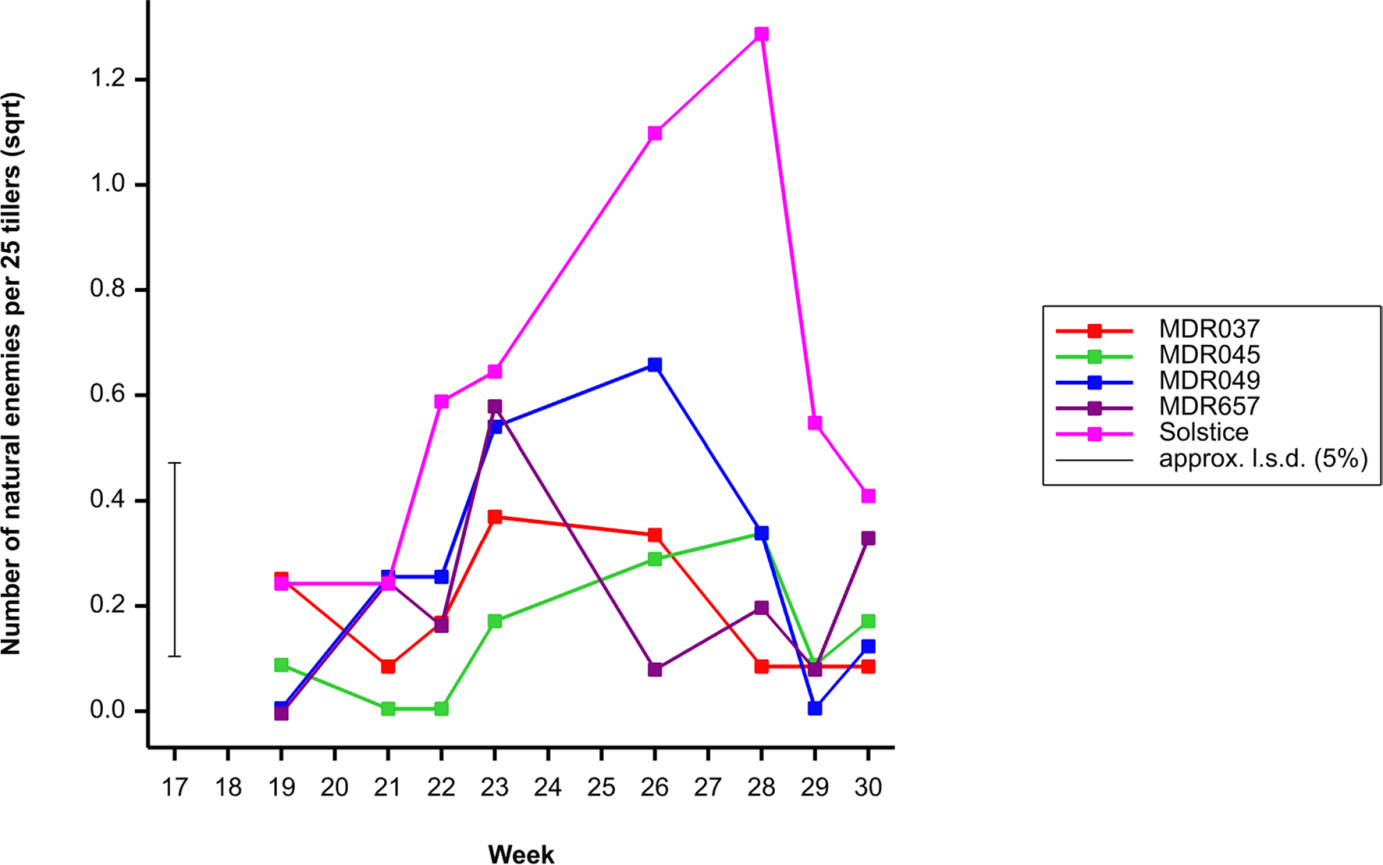
Average number of natural enemies (parasitised mummified aphids, ladybird adults and larvae, and Lacewing eggs and larvae) per 25 tillers observed on *Triticum aestivum* Solstice, *Triticum moncoccum* MDR037, MDR657, MDR045 and MDR049 from week 18 to week 30. All data was subject to square root transformation.

To determine whether any of the wheat genotypes conferred indirect resistance by attracting aphid natural enemies, natural enemy presence were investigated. The natural enemies investigated were parasitised mummified aphids, ladybirds adults and larvae and lacewing eggs and larvae which made up 64.7%, 23.5% and 11.8% of field natural enemy presence respectively. Due to low number the presence of these three insects were combined in analysis. The density of natural enemies differed between wheat genotypes (*F*_4, 599_ = 28.34, *P* < 0.001), the highest natural enemy presence per 25 tillers was observed on Solstice (Fig. 5). Natural enemy density varied by week (*F*_11, 599_ = 6.53, *P* < 0.001), the highest numbers were observed in weeks 24–28 (Fig. 5). There was no difference in natural enemy presence across the three field trial years (*F*_2, 599_ = 3.44, *P* = 0.052). There was, however, an interaction between the wheat genotype and week (*F*_44, 599_ = 1.88, *P* < 0.001) and week and year (*F*_16, 599_ = 2.12, *P* = 0.006). There was no interaction between the wheat genotype and year (*F*_8, 599_ = 1.88, *P* = 0.059) nor the wheat genotype, week and year (*F*_64, 599_ = 0.98, *P* = 0.519). The average number of natural enemies per 25 tillers across all three field trials is presented (Fig. 5).

To determine whether there was a relationship between the aphid populations and natural enemy presence observed throughout all three field trials, a simple linear regression was carried out.

**Figure 6:**
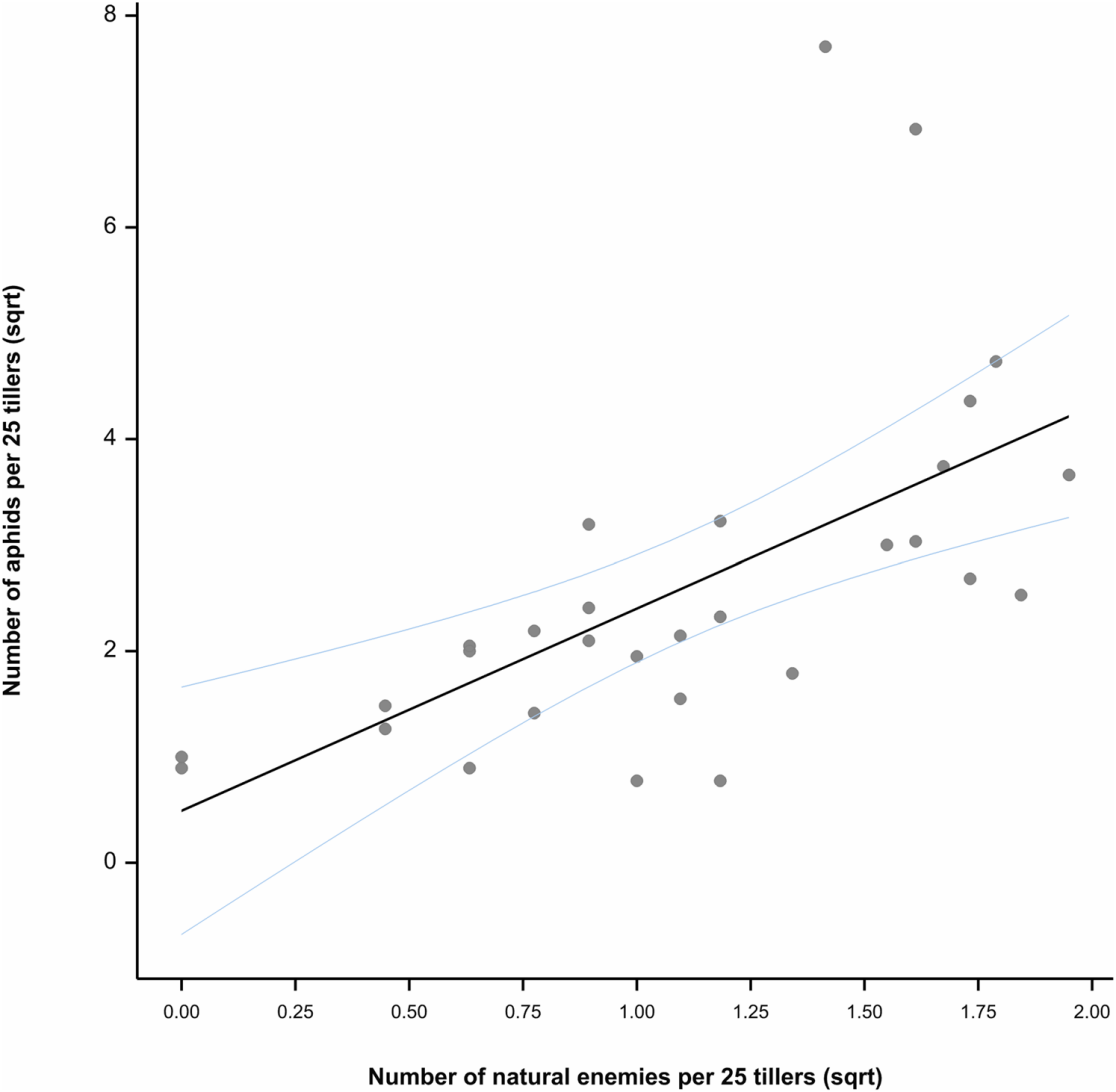
Correlation between the average number of aphids per 25 tillers and the average number of natural enemies per 25 tillers. Data was subject to square root transformation.

There was a positive relationship between the densities of aphids and natural enemies in the crop (*R*^2^= 0.28, *P* < 0.001) (Fig. 6).

### Temperature and rainfall during field trials

Data on temperature and rainfall across the three summer field trials was obtained from the Rothamsted Long-term Experiments National Capability (LTE-NCG) e-Rothamsted Archive. The highest temperatures and the least rainfall were recorded in 2018 (field trial year 2) (Table 1). Week averages of meteorological data are available in Supplementary Material.

**Table 1:** Average maximum temperature, minimum temperature, amount of rainfall and rainfall duration ± SD for May, June, and July during field trial year 1 (2017), 2 (2018) and 3 (2019) at Rothamsted Research, Harpenden, Hertfordshire, UK.

	Field trial year 1 (2017)			Field trial year 2 (2018)			Field trial year 3 (2019)		
	May	June	July	May	June	July	May	June	July
Maximum temperature (°C)	17.74 ± 4.21	21.52 ± 4.38	21.77 ± 3.03	18.67 ± 3.70	21.54 ± 3.27	26.06 ± 3.10	16.32 ± 3.16	19.30 ± 4.19	23.16 ± 4.08
Minimum temperature (°C)	8.65 ± 3.11	12.01 ± 2.43	13.21 ± 1.60	7.86 ± 3.39	10.87 ± 2.43	13.81 ± 1.55	6.34 ± 3.02	10.16 ± 2.81	12.97 ± 2.95
Rainfall (mm)	2.26 ± 5.06	1.29 ± 3.72	2.38 ± 4.78	2.00 ± 4.91	0.12 ± 0.28	0.49 ± 1.17	1.16 ± 2.44	2.86 ± 4.38	1.08 ± 2.33
Rainfall duration (h)	1.23 ± 2.64	0.75 ± 2.00	1.12 ± 2.20	0.79 ± 1.56	0.11 ± 0.24	0.28 ± 0.72	0.72 ± 1.36	1.32 ± 2.41	0.19 ± 0.43

Simple linear regressions were carried out to determine if observed aphid populations and presence of natural enemy densities were correlated with meteorological factors maximum temperature, minimum temperature, rainfall, and rainfall duration.

**Figure 7:**
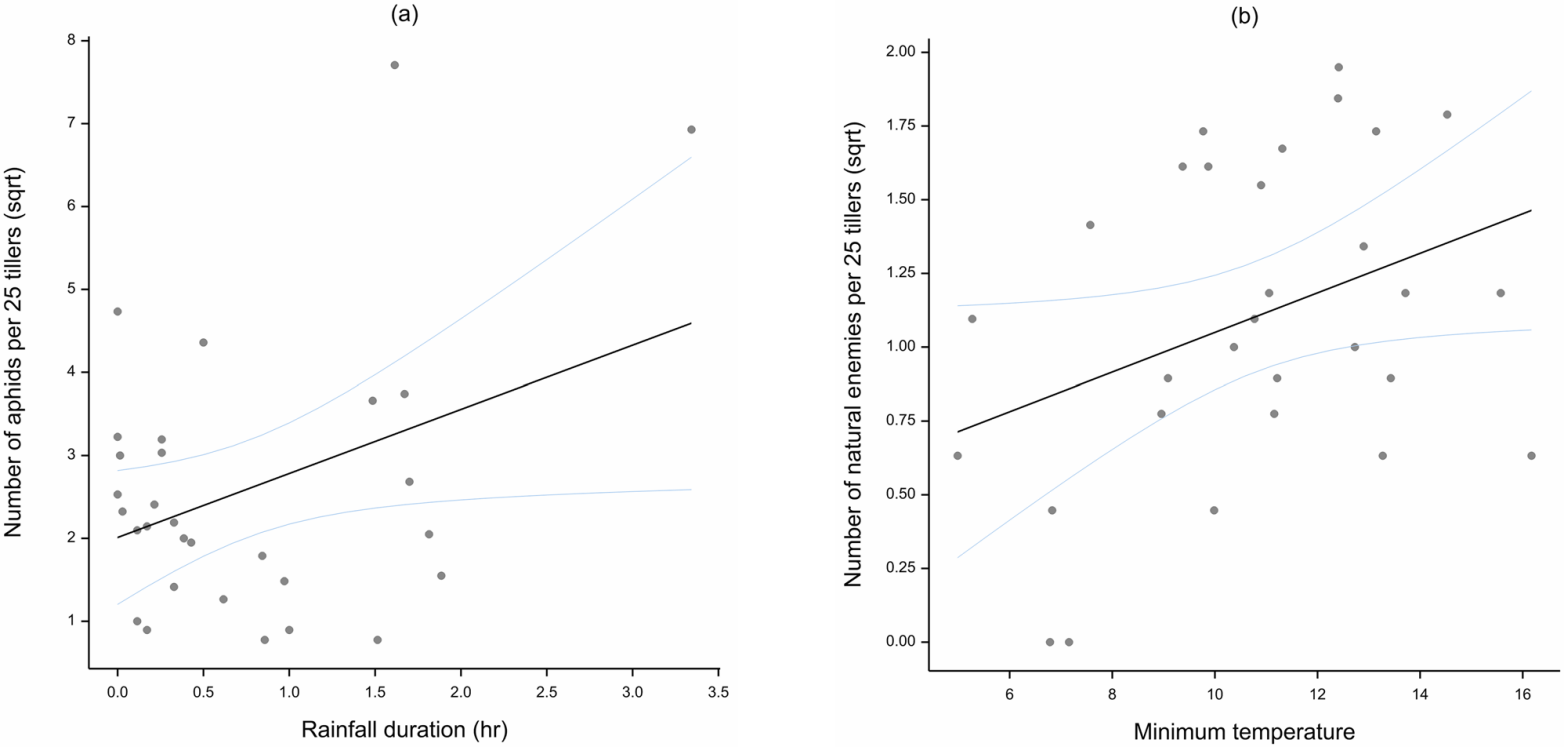
Correlation between (**a**) number of aphids per 25 tillers and rainfall duration (r) (**b**) number of natural enemies per 25 tillers and minimum temperature. The number of aphids and natural enemies per 25 tillers was subject to square root transformation.

There was a positive correlation between aphid density and rainfall duration (*R*^2^ = 0.109, *P* = 0.042) (Fig. 7a), and between natural enemy density and minimum temperature (*R*^2^ = 0.101, *P* = 0.048) (Fig. 7b). Correlations between aphids and natural enemies and all meteorological factors are presented in Supplementary Material.

## Discussion

Populations of aphids and natural enemies were recorded on five wheat genotypes over three years to test whether plant resistance to aphids observed in laboratory experiments would also hold under field conditions. The field trials confirmed natural resistance against the aphid species *Rhopalosiphum padi* and *Sitobion avenae* for *Triticum monococum* (L.) MDR045 and MDR049, identified resistance to *Metopolophium dirhodum* in *T. monococum* MDR045, and showed that natural enemy numbers were positively correlated with aphid densities. A consideration to factor in is the small size of the field plots (0.6–1 m^2^^2^) and ideally this kind of work will be repeated at larger field scale in the future.

Under laboratory conditions, antibiosis in *T. monococcum* has been shown to vary by genotype^33,37,38^. *Triticum monococcum* genotypes MDR657 and MDR049 have been shown to confer partial resistance to *R. padi*^33^*,* MDR045 and MDR049 have shown partial resistance to *S. avenae*^38^ and *R. padi* (Simon et al*.* unpublished)*.* Here we show that smaller populations of cereal aphid species, *R. padi* and *S. avenae* were also observed on MDR045 and MDR049 in field conditions, and for the first time demonstrating MDR045 confers resistance to *M. dirhodum*. However, as all aphid populations investigated on MDR657 were similar to those observed on the susceptible MDR037, it is concluded that this genotype did not confer resistance to these aphid species in field conditions.

Lower aphid populations observed on MDR045 and MDR049 could be due to direct and/or indirect resistance. Direct resistance can be via antixenosis or antibiosis, or both. Antixenosis relies on semiochemical and/or visual cues that prevent alate aphids alighting on the plant^24,26,42^. It has been shown that semiochemical production can vary between crop genotypes^43–45^ and that this can affect cereal aphid behaviour towards different crop genotypes^46^. The highest number of alate aphids was observed on Solstice, suggesting that this genotype was attractive to alate aphids and/or a consequence of antixenosis from *T. monococcum* genotypes. The role of semiochemicals in this interaction would be an important area for further research.

Antibiosis in plants causes reduced development and fecundity in aphids. As noted above, this type of resistance against aphids has been observed under laboratory conditions for MDR657, MDR045 and MDR049^33,38^. Smaller populations of all three aphid species were observed on MDR045 and smaller *R. padi* and *S. avenae* populations were observed on MDR049 in the field trials. It is suggested therefore that the resistance shown by MDR045 and MDR049 is antibiosis, making both these genotypes strong candidates for introgression into commercial wheat varieties to develop resistant plants as part of Integrated Pest Management strategies. During the introgression process it would be imperative to determine whether the reduced aphid populations would result in less virus transmission as well and consider any changes in yield and grain quality. This work confirms that the host genotype can affect aphid populations on these wheat lines under field conditions.

To determine whether any of the genotypes studied had indirect resistance (resistance mediated via another organism), natural enemy presence was observed. Natural enemies recorded included parasitised mummified aphids, ladybird adults and larvae and lacewing eggs and larvae. Overall, natural enemy densities were positively correlated with aphid densities in the field trials. This positive correlation has also been observed between ladybird and aphid densities^47^, and parasitised aphids and aphid densities^48^.

Surprisingly, natural enemy densities on MDR049 were comparable to their presence on MDR037 and MDR657. This indicates that MDR049 recruited more of these natural enemies in relation to its aphid population. It has previously been shown that natural enemies perform better on resistant plants; in a glasshouse study, lacewing *Chrysoperla plorabunda* (F.) larvae reduced aphid populations more on a resistant transgenic wheat line ^49^. It has also been shown that lacewing eggs on resistant plants had higher larval emergence and the proportion of female larvae increased on transgenic resistant *T. aestivum* than on the isogenic susceptible plants^50^. Therefore, it is hypothesised that the smaller aphid populations observed on MDR049 are due to a combination of slower aphid population growth, and more natural enemy presence and/or more effective parasitism. To further investigate natural enemy population effects on MDR049, cage studies where natural enemies can be controlled should be carried out in laboratory and/or field conditions.

Aphid populations varied between years, the lowest number of aphids was observed in the second field trial year, 2018, which had the highest average maximum and minimum temperature, and lowest rainfall duration and average rainfall in June and July. The UK Meteorological Office declared a heatwave during the second field trial year. A heatwave is “A marked unusual hot weather (Max, Min and daily average) over a region persisting at least two consecutive days during the hot period of the year based on local climatological conditions, with thermal conditions recorded above given thresholds.” (*Guidelines on the Defintion and Monitoring of Extreme Weather and Climate Events*, 2016). Whilst aphid populations were not correlated with temperature, there was a positive correlation between aphid population densities and rainfall duration.

Within the *T. monococcum* genotypes, MDR037 had the highest number of aphids in the first and third year of field trials. MDR037 has also been shown to be susceptible to *R. padi* and *S. avenae* infestation under controlled conditions^33,38^. However, in the second year, MDR037 had the second lowest aphid population and a lower aphid presence than MDR049. The reduced rainfall was correlated with reduced aphid population densities, although it seems that aphids on MDR037 were more adversely affected by the heatwave conditions than those on the other genotypes. Drought stress has previously been shown to affect aphid feeding behaviour, for example in an experiment where *R. padi* was feeding on drought stressed *Hordeum vulgare,* the aphids had a lower proportion of probes where phloem feeding occurred and salivated for longer than those on well-watered plants^51^. In another study on drought stressed *H. vulgare,* the greenbug (*Schizaphis Graminum* (Rondani)) had slower development rates than those on well-watered plants^52^. It has also been shown that *R. padi* and *S. avenae,* on drought-stressed *T. aestivum* produced fewer nymphs and had a lower intrinsic rate of increase^53^. Therefore, it is hypothesised that drought stress reduced MDR037 susceptibility as an aphid host and that drought differentially affects the *T. monococcum* genotypes.

Natural enemy presence was positively correlated with minimum temperature; however, it is unknown how much this is impacted by aphids and other factors. A number of studies have reported positive correlations between temperature and a natural enemy; temperature has a positive effect on the development rates of the lacewing *Chrysopodes lineafrons* (A.P.)^54^, and the parasitoid *Aphidus ervi* (Haliday)^55^. Temperature can also influence predator success, as in the example of the aphid endosymbiont *Hamiltonella defensa* (M.) that has been shown to provide protection against parasitism against *A. ervi* at 20 °C was reduced at 25 °C and aphids were susceptible to parasitism at 30 °C^56,57^. Therefore, increased natural enemy presence observed at higher temperatures may be due to a combination of reduced aphid defences against parasitism as well as increased parasitoid and lacewing development. Climate change scenarios predict more frequent and intense summer droughts^58^, leading to increased aphid and parasitoid abundance^17^. Here we show that aphid densities were positively correlated with rainfall duration, and natural enemy presence can increase with higher temperatures. These meteorological interactions should be further investigated to better understand the impacts of climate change on wheat-aphid-natural enemy interactions.

We have shown that aphid population densities were dependent on wheat genotype, confirming that the partial aphid resistance to *R. padi* and *S. avenae* observed in *T. monococcum* MDR045 and MDR049 under laboratory conditions, are also present under field conditions. We also show for the first time that *T. monococcum* MDR045 confers resistance to *M. dirhodum.* In addition to reduced aphid population growth, MDR049 also recruited natural enemies although it is unknown how much the predation and/or parasitism impacted the observed aphid populations.

## Materials and methods

### Plant material

*Triticum aestivum* seeds were provided by Rothamsted Research, Hertfordshire, UK. *Triticum monococcum* MDR037, MDR657, MDR045 and MDR049 seeds were provided by the Wheat Genetic Improvement Network (WGIN). All plants used in these field trials complied with international, national and institutional guidelines.

### Field trials experimental design

Observations of aphid and natural enemy presence on *Triticum monococcum* genotypes MDR037, MDR657, MDR045, MDR049 and *Triticum aestivum* cv. Solstice were carried out at Rothamsted Research, Hertfordshire, UK. Seeds were sown in Autumn at 250 seeds per plot for the first field trial in 2017, and 300 seeds per plot for the second and third field trials in 2018 and 2019 respectively. The first field trial was sown in a field named Long Hoos 4, in 0.6 × 1 m plots sown at a drill speed of 0.7miles per hour (mph), plots were separated by 0.5 m unsown ground. The second and third field trials were at Long Hoos 5 and Claycroft respectively, with 1 m^2^ plots sown at a drill speed of 1.2mph, plots were separated by 0.75 m unsown ground. The reason for the smaller plot size in the first year was due to a limited availability of seed. Subsequent years used saved seeds hand harvested from previous field season. The plots were sown in a randomised block design with four replicate plots for each genotype. At each side of the field trial was a strip of *T. aestivum* var. Hereward.

### Measuring aphid populations and natural enemy presence

The number of cereal aphids (*Sitobion avenae*, *Rhopalosiphum padi* and *Metopolophium dirhodum*), and natural enemies (ladybird adults and larvae, lacewing eggs and larvae and parasitised mummified aphids) per tiller was counted on 25 tillers in each plot on a weekly or biweekly basis, depending on weather. Each tiller was chosen at random using Genstat (2016, 18th Edition, VSN International Ltd, Hemel Hempstead, UK) statistical software. The sampling started either the first or second week of May when the aphid populations started to increase and finished in the last week of July when aphid populations had diminished.

Meteorological data on daily maximum temperature, minimum temperature, and rainfall were provided by Rothamsted Research Long-term Experiments National Capabilities (LTE-NCG) Electronic Rothamsted Archive (e-RA).

### Statistical analysis

Data were subjected to transformation to comply with assumptions of normality and equal variance. Normailty was determined through the Shapiro–Wilk test and equal variances through the homogeneity of variance test. The number of *R. padi*, *S. avenae*, *M. dirhodum*, alate aphids and natural enemy presence per 25 tillers were subject to square root transformation. Genstat software (19th Edition, VSN International Ltd, Hemel Hempstead, UK) was used for the following analysis. The number of *R. padi*, *S. avenae*,* M. dirhodum*, alate aphids and natural enemy presence per 25 tillers was analysed using linear mixed model (LMM) form of the restricted maximum likelihood model (REML) as there were some missing values. The wheat genotype, field trial year and the week of recording were the fixed model. The random model included blocks of the field trial design and the time of assessments.

To determine relationships between aphid populations and natural enemy presence, a simple linear regression was carried out. Relationships between weather conditions and aphid or natural insect presence were determined through simple linear regression analysis.

### Ethical approval

This article does not contain any studies on human participants or cells performed by any of the authors.

## Supplementary Information

Supplementary Material.

## Data Availability

All original data within this manuscript is available in FigShare, https://figshare.com/.
