# Supplementary material for: Identifying aphid resistance in the ancestral wheat Triticum monococcum under field conditions
Source: Sci Rep. 2021 Jun 29;11:13495. doi: 10.1038/s41598-021-92883-9 (PMC8241983; doi:10.1038/s41598-021-92883-9)
Supplement: Supplementary file 1 — Supplementary Material. [file 41598_2021_92883_MOESM1_ESM.docx]

**Supplementary material**

To determine interactions between different variables within the field trials, post hoc analysis on the effect of *Triticum* genotype, field trial year and week on *Rhopalosiphum padi, Sitobion avenae, Metopolophium dirhodum,* alate aphids and natural enemy presence per 25 tillers was carried out. Due to the restraints of Genstat statistical software used for initial REML analysis, analysis for post hoc differences between variables were done in R (R Core Team (2021). R Foundation for Statistical Computing, Vienna, Austria) using the lme4 and multcomp packages.

*Triticum aestivum* Solstice had higher populations of all insects than the *Triticum monococcum* genotypes.

*Triticum monococcum* MDR045 had smaller *R. padi* populations than Solstice (Z = 5.48, *P* <0.001) and MDR037 (Z = 2.85, *P* = 0.031), smaller *S. avenae* populations than Solstice (Z = 9.29, *P* = <0.001), MDR037 (Z = 3.47, *P* = 0.003) and MDR657 (Z = 3.34, *P* = 0.004), and smaller *M. dirhodum* populations than Solstice (Z = 6.68, *P* <0.001) and MDR037 (Z = 6.68, *P* = 0.021).

*Triticum monococcum* MDR049 had smaller *R. padi* populations than Solstice (Z = 5.48, *P* <0.001) and MDR037 (Z = 2.85, *P* = 0.031), and smaller *S. avenae* populations than Solstice (Z = 8.67, *P* <0.001) and MDR037 (Z = 2.84, *P* = 0.018).

Natural enemy presence on MR049 was smaller than on MDR045 (Z = 2.94, *P* = 0.019).

**Table 1.** Post hoc analysis on the effect of *Triticum* genotype on *Rhopalosiphum padi, Sitobion avenae, Metopolophium dirhodum,* alate aphids and natural enemy presence per 25 tillers observed on *Triticum aestivum* Solstice, *Triticum monococcum* MDR037, MDR657, MDR045 and MDR049 in field trials.

| Hypothesis | *Rhopalosiphum padi* | | *Sitobion avenae* | | *Metopolophium dirhodum* | | Alate aphids | | Natural enemy presence | |
| --- | --- | --- | --- | --- | --- | --- | --- | --- | --- | --- |
| Genotype | Z value | P | Z value | P | Z value | P | Z value | P | Z value | P |
| MDR045 – MR037 = 0 | -2.846 | 0.031 * | -3.468 | 0.003 ** | -2.919 | 0.021 * | 0.631 | 1.00 | -1.377 | 0.5888 |
| MDR049 – MR037 = 0 | -2.846 | 0.031* | -2.842 | 0.018 * | -1.979 | 0.191 | 0.421 | 1.00 | 1.564 | 0.5888 |
| MDR657 – MR037 = 0 | -1.687 | 0.367 | -0.130 | 1.00 | -0.396 | 0.694 | 0.842 | 1.00 | 0.140 | 0.8890 |
| Solstice – MR037 = 0 | 2.637 | 0.042 * | 5.826 | <0.001 *** | 3.761 | 0.001** | 7.790 | <0.001 *** | 13.512 | <0.001 *** |
| MDR049 – MR045 = 0 | 0.00 | 1.00 | 0.626 | 1.00 | 0.940 | 0.694 | -0.210 | 1.00 | 2.938 | 0.02 * |
| MDR657 – MR045 = 0 | 1.160 | 0.739 | 3.337 | 0.004 ** | 2.524 | 0.058 | 0.210 | 1.00 | 1.514 | 0.5888 |
| Solstice – MDR045 = 0 | 5.484 | <0.001 *** | 9.294 | <0.001 *** | 6.680 | <0.001 *** | 7.158 | <0.001 *** | 14.875 | <0.001 *** |
| MDR657 – MDR049 = 0 | 1.160 | 0.739 | 2.712 | 0.020 * | 1.583 | 0.340 | 0.421 | 1.00 | -1.426 | 0.5888 |
| Solstice – MDR049 = 0 | 5.484 | <0.001 *** | 8.668 | <0.001 *** | 5.740 | <0.001 *** | 7.369 | <0.001 *** | 11.917 | <0.001 *** |
| Solstice – MDR657 = 0 | 4.324 | <0.001 *** | 5.957 | <0.001 *** | 4.157 | <0.001 *** | 6.948 | <0.001 *** | 13.360 | <0.001 *** |

Field trial year 2018 had smaller *R. padi* populations than years 2017 (Z = 3.66, *P* < 0.001) and 2019 (Z = 5.24, *P* < 0.001), and smaller *M. dirhodum* populations than years 2017 (Z = 2.65, *P* = 0.016) and 2019 (Z = 4.13, *P* <0.001). Field trial 2019 had higher *S. avenae* than years 2017 (Z = 5.78, *P* <0.001) and 2018 (Z = 6.61, *P* <0.001). Alate aphid and natural enemy presence was the same for all three field trial years.

**Table 2**. Post hoc analysis on the effect of field trial year on *Rhopalosiphum padi, Sitobion avenae, Metopolophium dirhodum,* alate aphids and natural enemy presence per 25 tillers observed during field trial years 1 (2017), 2 (2018) and 3 (2019).

| Hypothesis | *Rhopalosiphum padi* | | *Sitobion avenae* | | *Metopolophium dirhodum* | | Alate aphids | | Natural enemy presence | |
| --- | --- | --- | --- | --- | --- | --- | --- | --- | --- | --- |
| Year | Z value | P | Z value | P | Z value | P | Z value | P | Z value | P |
| 2018 - 2017 = 0 | -3.661 | <0.001 *** | -0.949 | 0.342 | -2.651 | 0.016 * | -0.404 | 0.686 | -0.313 | 0.754 |
| 2019 - 2017 = 0 | 1.539 | 0.124 | 5.784 | <0.001 *** | 1.464 | 0.143 | 1.834 | 0.133 | -2.294 | 0.065 |
| 2019 - 2018 = 0 | 5.242 | <0.001 *** | 6.614 | <0.001 *** | 4.134 | <0.001 *** | 2.200 | 0.083 | -1.915 | 0.111 |

Week 23 and 27 had the highest populations of *R. padi,* with higher populations that 10 and 5 other weeks respectively. Week 23 and 24 has higher *S. avenae* populations than 9 and 11 other weeks respectively. Week 23 had higher *M. dirhodum* than 11 other weeks. Week 23 also had higher alate aphid populations than 10 other weeks. Weeks 24 – 28 had higher natural enemy populations than 7 other weeks.

**Table 3.** Post hoc REML analysis on the effect of the week on *Rhopalosiphum padi, Sitobion avenae, Metopolophium dirhodum,* alate aphids and natural enemy presence per 25 tillers observed during field trial weeks 18 to 30.

| Hypothesis | *Rhopalosiphum padi* | | *Sitobion avenae* | | *Metopolophium dirhodum* | | Alate aphids | | Natural enemy presence | |
| --- | --- | --- | --- | --- | --- | --- | --- | --- | --- | --- |
| Week | Z value | P | Z value | P | Z value | P | Z value | P | Z value | P |
| 19 – 18 = 0 | 0.268 | 1.00 | 0.155 | 1.00 | -0.021 | 1.00 | -0.909 | 1.00 | 0.624 | 1.00 |
| 20 – 18 = 0 | 1.013 | 1.00 | 0.598 | 1.00 | 0.231 | 1.00 | 0.916 | 1.00 | 0.396 | 1.00 |
| 21 – 18 = 0 | 0.389 | 1.00 | 0.809 | 1.00 | 0.133 | 1.00 | -0.094 | 1.00 | 1.044 | 1.00 |
| 22 – 18 = 0 | 0.811 | 1.00 | 1.026 | 1.00 | 1.470 | 1.00 | -0.901 | 1.00 | 1.878 | 1.00 |
| 23 – 18 = 0 | 4.822 | <0.001 *** | 6.098 | <0.001 *** | 5.186 | <0.001 *** | 3.511 | 0.025* | 3.840 | 0.004 ** |
| 24 – 18 = 0 | 2.063 | 1.00 | 8.721 | <0.001 *** | 2.442 | 0.613 | -0.094 | 1.00 | 4.841 | <0.001 *** |
| 26 – 18 = 0 | 2.394 | 1.00 | 4.227 | <0.001 *** | 2.858 | 0.196 | 0.904 | 1.00 | 5.523 | <0.001 *** |
| 27 – 18 = 0 | 3.708 | 1.00 | 2.763 | 0.206 | 0.828 | 1.00 | -1.234 | 1.00 | 4.841 | <0.001 *** |
| 28 – 18 = 0 | 2.183 | 1.00 | 1.189 | 1.00 | 0.827 | 1.00 | 0.503 | 1.00 | 4.681 | <0.001 *** |
| 29 – 18 = 0 | 0.389 | 1.00 | 0.999 | 1.00 | 0.083 | 1.00 | -1.301 | 1.00 | 1.325 | 1.00 |
| 30 – 18 = 0 | -0.244 | 1.00 | 0.050 | 1.00 | -0.263 | 1.00 | -1.904 | 1.00 | 1.597 | 1.00 |
| 20 – 19 = 0 | 0.986 | 1.00 | 0.586 | 1.00 | 0.309 | 1.00 | 2.092 | 1.00 | -0.157 | 1.00 |
| 21 – 19 = 0 | 0.175 | 1.00 | 0.948 | 1.00 | 0.222 | 1.00 | 1.204 | 1.00 | 0.628 | 1.00 |
| 22 – 19 = 0 | 0.787 | 1.00 | 1.262 | 1.00 | 2.159 | 1.00 | 0.007 | 1.00 | 1.851 | 1.00 |
| 23 – 19 = 0 | 6.594 | <0.001 *** | 8.605 | <0.001 *** | 7.538 | <0.001 *** | 6.448 | <0.001 *** | 4.745 | <0.001 *** |
| 24 – 19 = 0 | 2.478 | 1.00 | 11.743 | <0.001 *** | 3.371 | 0.039 * | 1.057 |  | 5.930 | <0.001 *** |
| 26 – 19 = 0 | 3.079 | 1.00 | 5.896 | <0.001 *** | 4.167 | 0.002** | 2.642 | 0.396 | 7.227 | <0.001 *** |
| 27 – 19 = 0 | 4.732 | <0.001 *** | 3.585 | 0.013 * | 1.160 | 1.00 | -0.519 | 1.00 | 5.930 | <0.001 *** |
| 28 – 19 = 0 | 2.773 | 1.00 | 1.498 | 1.00 | 1.226 | 1.00 | 0.2056 | 1.00 | 5.986 | <0.001 *** |
| 29 – 19 = 0 | 0.175 | 1.00 | 1.223 | 1.00 | 0.150 | 1.00 | -0.580 | 1.00 | 1.049 | 1.00 |
| 30 – 19 = 0 | -0.742 | 1.00 | -0.151 | 1.00 | -0.352 | 1.00 | -1.457 | 1.00 | 1.435 | 1.00 |
| 21 – 20 = 0 | -0.861 | 1.00 | 0.083 | 1.00 | -0.152 | 1.00 | -1.254 | 1.00 | 0.602 | 1.00 |
| 22 – 20 = 0 | -0.430 | 1.00 | 0.304 | 1.00 | 1.212 | 1.00 | -2.105 | 1.00 | 1.490 | 1.00 |
| 23 – 20 = 0 | 3.659 | 0.013 * | 5.475 | <0.001 *** | 5.000 | <0.001 *** | 2.526 | 0.542 | 3.575 | 0.012 * |
| 24 – 20 = 0 | 0.913 | 1.00 | 8.173 | <0.001 *** | 2.214 | 1.00 | -1.191 | 1.00 | 4.635 | <0.001 *** |
| 26 – 20 = 0 | 1.184 | 1.00 | 3.567 | 0.013 * | 2.626 | 0.388 | -0.221 | 1.00 | 5.363 | <0.001 *** |
| 27 – 20 = 0 | 2.587 | 1.00 | 2.112 | 1.00 | 0.571 | 1.00 | -2.382 0.744707 | 1.00 | 4.635 | <0.001 *** |
| 28 – 20 = 0 | 0.969 | 1.00 | 0.470 | 1.00 | 0.556 | 1.00 | -0.632 | 1.00 | 4.469 | <0.001 *** |
| 29 – 20 = 0 | -0.861 | 1.00 | 0.277 | 1.00 | -0.202 | 1.00 | -2.514 | 0.549 | 0.899 | 1.00 |
| 30 – 20 = 0 | -1.507 | 1.00 | -0.691 | 1.00 | -.0556 | 1.00 | -3.158 | 0.084 | 1.192 | 1.00 |
| 22 – 21 = 0 | 0.609 | 1.00 | -0.313 | 1.00 | 1.929 | 1.00 | -1.192 |  | 1.247 | 1.00 |
| 23 – 21 = 0 | 6.392 | <0.001 *** | 7.626 | <0.001 *** | 7.286 | <0.001 *** | 5.323 | <0.001 *** | 4.179 | 0.001 ** |
| 24 – 21 = 0 | 2.314 | 1.00 | 10.860 | <0.001 *** | 3.162 | 0.075 | -0.007 | 1.00 | 5.430 | <0.001 *** |
| 26 – 21 = 0 | 2.892 | 1.00 | 4.927 | <0.001 *** | 3.928 | 0.005** | 1.473 | 1.00 | 6.692 | <0.001 *** |
| 27 – 21 = 0 | 4.560 | <0.001 *** | 2.728 | 0.222 | 0.958 | 1.00 | -1.598 | 1.00 | 5.430 | <0.001 *** |
| 28 – 21 = 0 | 2.587 | 1.00 | 0.547 | 1.00 | 1.00 | 1.00 | 0.881 | 1.00 | 5.435 | <0.001 *** |
| 29 – 21 = 0 | 0.00 | 1.00 | 0.274 | 1.00 | -0.071 | 1.00 | -1.786 | 1.00 | 0.421 | 1.00 |
| 30 – 21 = 0 | -0.913 | 1.00 | -1.095 | 1.00 | -0.571 | 1.00 | -2.673 | 0.377 | 0.827 | 1.00 |
| 23 – 22 = 0 | 5.783 | <0.001 *** | 7.313 | <0.001 *** | 5.357 | <0.001 *** | 6.549 | <0.001 *** | 2.949 | 0.102 |
| 24 – 22 = 0 | 1.770 | 1.00 | 10.580 | <0.001 *** | 1.437 | 1.00 | 1.065 | 1.00 | 4.333 | <0.001 *** |
| 26 – 22 = 0 | 2.283 | 1.00 | 4.614 | <0.001 *** | 2.00 | 1.00 | 2.679 | 0.377 | 5.477 | <0.001 *** |
| 27 – 22 = 0 | 4.016 | 0.003** | 2.448 | 0.459 | -0.767 | 1.00 | -0.533 | 1.00 | 4.333 | <0.001 *** |
| 28 – 22 = 0 | 1.978 | 1.00 | 0.235 | 1.00 | -0.292 | 1.00 | 2.084 | 1.00 | 4.213 | <0.001 *** |
| 29 – 22 = 0 | -0.609 | 1.00 | -0.039 | 1.00 | 2.00 | 1.00 | -0.585 | 1.00 | -0.828 | 1.00 |
| 30 – 22 = 0 | -1.522 | 1.00 | -1.408 | 1.00 | -2.500 | 0.534 | -1.489 | 1.00 | -0.422 | 1.00 |
| 24 - 23 = 0 | -3.403 | 0.033 * | 4.040 | 0.002** | -3.354 | 0.040* | -4.793 | <0.001 *** | 1.696 | 1.00 |
| 26 - 23 = 0 | -3.500 | 0.024* | -2.698 | 0.237 | -3.357 | 0.040* | -3.870 | <0.001 *** | 2.528 | 0.356 |
| 27 - 23 = 0 | -1.157 | 1.00 | -4.092 | 0.002 ** | -5.558 | <0.001 *** | -6.391 | <0.001 *** | 1.696 | 1.00 |
| 28 - 23 = 0 | -3.805 | 0.008** | -7.078 | <0.001 *** | -6.286 | <0.001 *** | -4.466 | <0.001 *** | 1.264 | 1.00 |
| 29 - 23 = 0 | -6.392 | <0.001 *** | -7.352 | <0.001 *** | -7.357 | <0.001 *** | -7.100 | <0.001 *** | -3.760 | 0.006 ** |
| 30 - 23 = 0 | -7.305 | <0.001 *** | -8.720 | <0.001 *** | -7.857 | <0.001 *** | -8.038 | <0.001 *** | -3.371 | 0.025 * |
| 26 – 24 = 0 | 0.272 | 1.00 | -6.453 | <0.001 *** | 0.351 | 1.00 | 1.331 | 1.00 | 0.565 | 1.00 |
| 27 – 24 = 0 | 2.050 | 1.00 | -7.424 | <0.001 *** | -2.012 | 1.00 | -1.459 | 1.00 | 0.00 | 1.00 |
| 28 – 24 = 0 | 0.00 | 1.00 | -10.371 | <0.001 *** | -2.268 | 0.957 | 0.799 | 1.00 | -0.565 | 1.00 |
| 29 – 24 = 0 | -2.314 | 1.00 | -10.615 | <0.001 *** | -3.226 | 0.061 | -1.584 | 1.00 | -5.055 | <0.001 *** |
| 30 – 24 = 0 | -3.131 | 1.00 | -11.840 | <0.001 *** | -3.673 | 0.013* | -2.397 | 0.745 | -4.711 | <0.001 *** |
| 27 – 26 = 0 | 1.974 | 1.00 | -1.679 | <0.001 *** | -2.555 | 0.467 | -2.929 | 0.177 | -0.565 | 1.00 |
| 28 – 26 = 0 | -0.304 | 1.00 | -4.380 | <0.001 *** | -2.928 | 0.160 | -0.595 | 1.00 | -1.264 | 1.00 |
| 29 – 26 = 0 | -2.892 | 1.00 | -4.654 | <0.001 *** | -4.000 | 0.004** | -3.250 | 0.062 | -6.273 | <0.001 *** |
| 30 – 26 = 0 | -3.805 | 0.008 ** | -6.022 | <0.001 *** | -4.500 | <0.001 *** | -4.168 | 0.002** | -5.899 | <0.001 *** |
| 28 – 27 = 0 | -2.246 |  | -2.239 | 0.781 | -0.064 | 1.00 | 2.397 | 0.745 | -0.565 | 1.00 |
| 29 – 27 = 0 | -4.560 | <0.001 *** | -2.483 | 0.430 | -1.022 | 1.00 | 0.007 | 1.00 | -5.055 | <0.001 *** |
| 30 – 27 = 0 | -5.377 | <0.001 *** | -3.708 | 0.008 ** | -1.469 | 1.00 | -0.799 | 1.00 | -4.711 | <0.001 *** |
| 29 – 28 = 0 | -2.587 | 1.00 | -0.274 | 1.00 | -1.071 | 1.00 | -2.658 | 0.386 | -5.016 | <0.001 *** |
| 30 – 28 = 0 | -3.500 | 0.024* | -1.642 | 1.00 | -1.571 | 1.00 | -3.573 | 0.020 * | -4.635 | <0.001 *** |
| 30 - 29 = 0 | -0.913 | 1.00 | -1.369 | 1.00 | -0.500 | 1.00 | -0.896 | 1.00 | 0.408 | 1.00 |

Daily meteorological parameters of maximum temperature, minimum temperature, rainfall (mm) and rainfall duration (hr) were Rothamsted Research Long-term Experiments National Capabilities (LTE-NCG) Electronic Rothamsted Archive (e-RA). A two-factor ANOVA was done on this data with the field trial year and week as factors.

**Table 4.** Average maximum temperature, minimum temperature, amount of rainfall and rainfall duration ± SE for each week during field year 1 (2017), 2 (2018) and 3 (2019) at Rothamsted Research, Harpenden, Hertfordshire, UK.

| **Year** | **Week** | **Maximum temperature** | **Minimum temperature** | **Rainfall (mm)** | **Rainfall duration (hr)** |
| --- | --- | --- | --- | --- | --- |
| 2017 | 18 | 13.93 ±0.73 | 6.79 ±0.52 | 0.14 ±0.14 | 0.11 ±0.11 |
|  | 19 | 15.83 ±1.53 | 6.83 ±1.56 | 0.69 ±0.30 | 0.62 ±0.23 |
|  | 21 | 23.10 ±0.62 | 10.37 ±0.55 | 1.00 ±0.93 | 0.43 ±0.36 |
|  | 22 | 20.17 ±0.73 | 11.21 ±0.78 | 0.56 ±0.42 | 0.26 ±0.17 |
|  | 23 | 18.46 ±0.97 | 11.31 ±0.84 | 3.47 ±2.28 | 1.67 ±0.97 |
|  | 26 | 25.39 ±1.73 | 14.53 ±0.75 | 0.00 ±0.00 | 0.00 ±0.00 |
|  | 27 | 19.29 ±1.03 | 11.86 ±0.70 | 1.93 ±1.70 | 1.41 ±1.18 |
|  | 28 | 20.99 ±0.81 | 13.14 ±0.54 | 3.09 ±2.81 | 1.70 ±1.50 |
|  | 29 | 21.24 ±1.11 | 13.27 ±0.77 | 3.94 ±1.44 | 1.81 ±0.60 |
|  | 30 | 19.50 ±0.54 | 12.73 ±0.21 | 3.49 ±2.09 | 1.51 ±0.81 |
| 2018 | 19 | 18.71 ±1.60 | 7.16 ±0.69 | 1.34 ±1.07 | 1.00 ±0.76 |
|  | 21 | 20.74 ±1.21 | 9.99 ±0.90 | 2.59 ±1.18 | 0.97 ±0.36 |
|  | 22 | 20.59 ±1.24 | 12.90 ±0.58 | 3.56 ±3.52 | 0.84 ±0.81 |
|  | 23 | 18.49 ±0.73 | 10.77 ±0.78 | 0.17 ±0.09 | 0.17 ±0.09 |
|  | 24 | 19.77 ±0.84 | 9.87 ±0.87 | 0.30 ±0.19 | 0.26 ±0.15 |
|  | 26 | 26.74 ±0.47 | 10.90 ±0.38 | 0.00 ±0.00 | 0.01 ±0.01 |
|  | 28 | 24.43 ±1.18 | 12.40 ±0.27 | 0.00 ±0.00 | 0.00 ±0.00 |
|  | 29 | 25.47 ±0.56 | 13.71 ±0.46 | 0.03 ±0.03 | 0.03 ±0.03 |
|  | 30 | 27.54 ±1.79 | 15.57 ±0.40 | 1.47 ±0.73 | 0.86 ±0.49 |
| 2019 | 19 | 13.90 ±0.60 | 5.27 ±0.91 | 3.17 ±1.70 | 1.89 ±0.93 |
|  | 20 | 16.33 ±0.55 | 4.99 ±0.93 | 0.44 ±0.30 | 0.39 ±0.25 |
|  | 21 | 19.83 ±0.47 | 9.09 ±0.98 | 0.24 ±0.24 | 0.11 ±0.11 |
|  | 22 | 20.09 ±1.42 | 8.96 ±0.98 | 0.64 ±0.43 | 0.33 ±0.14 |
|  | 23 | 17.31 ±0.55 | 7.57 ±0.72 | 1.87 ±0.88 | 1.61 ±0.70 |
|  | 24 | 15.34 ±1.06 | 9.37 ±0.36 | 4.73 ±2.43 | 3.34 ±1.45 |
|  | 26 | 23.04 ±1.79 | 13.67 ±0.80 |  | 0.00 ±0.00 |
|  | 27 | 21.66 ±0.80 | 11.06 ±0.94 | 0.00 ±0.00 | 0.00 ±0.00 |
|  | 28 | 22.24 ±0.63 | 13.43 ±0.79 | 0.00 ±0.00 | 0.21 ±0.21 |
|  | 29 | 22.13 ±0.75 | 11.16 ±1.04 | 1.68 ±1.30 | 0.33 ±0.24 |
|  | 30 | 27.14 ±2.45 | 16.17 ±0.66 | 1.73 ±0.81 | 0.17 ±0.08 |

To determine whether correlations between meteorological factors maximum temperature, minimum temperature, rainfall and rainfall duration and aphids differ between the three aphid species, linear regressions were carried out on *R. padi, S. avenae,* *M. dirhodum* as well as overall aphid densities. Analysis was done using Genstat (2016, 19^th^ Edition, VSN International Ltd, Hemel Hempstead, UK).

There was no correlation between aphid population densities and maximum temperature (*R^2^*= 0.166, *P*= 0.411), minimum temperature (*R^2^*= 0.168, *P*= 0.695) or rainfall (*R^2^*= 0.165, *P*= 0.323), however, there was a positive correlation between aphid density and rainfall duration (*R^2^*= 0.109, *P*= 0.042) (Fig 7).


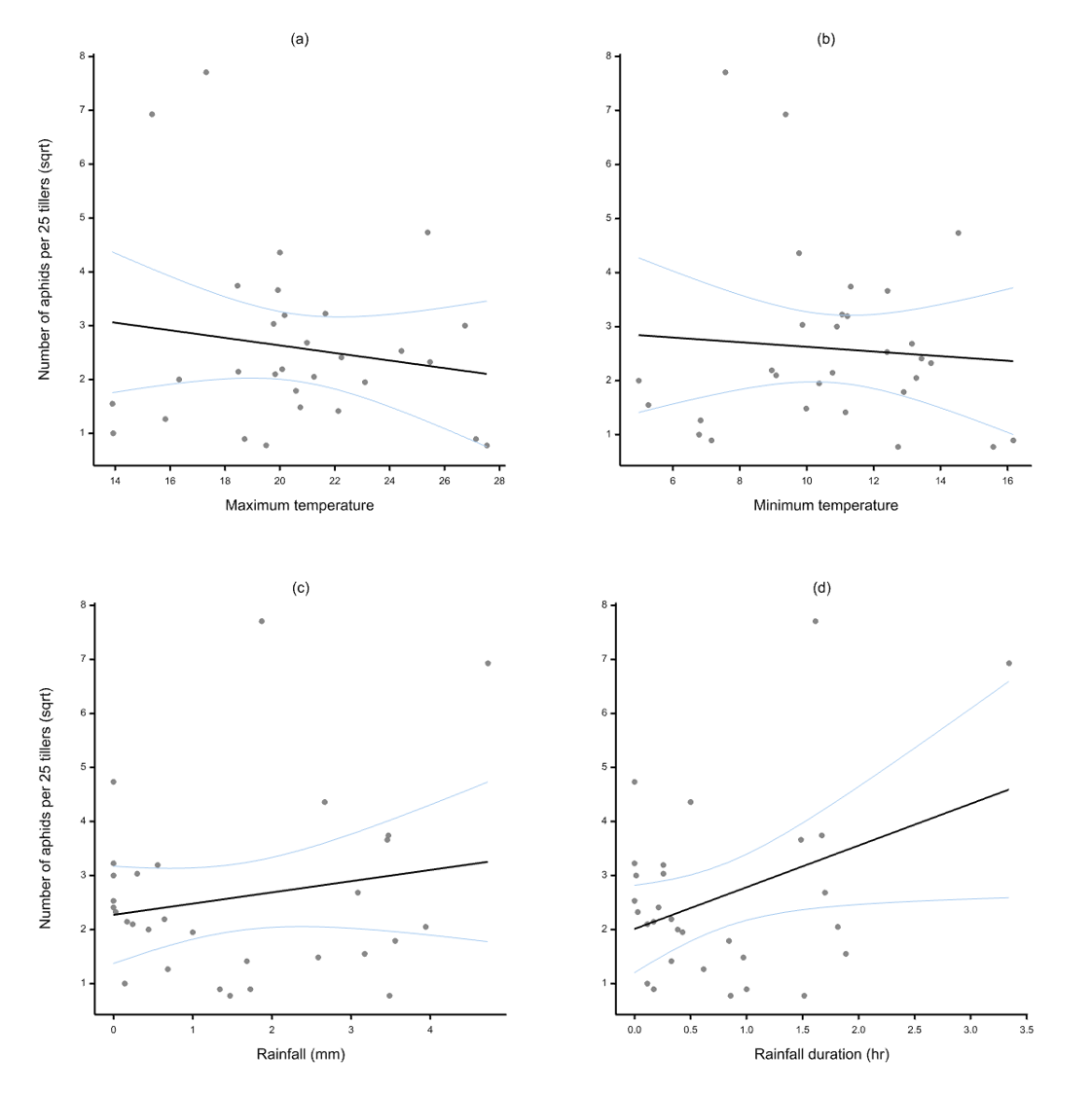


**Figure 1.** Correlation between number of aphids per 25 tillers and meteorological factors **a)** maximum temperature, **b)** minimum temperature, **c)** rainfall (mm), **d)** rainfall duration (hr). Number of natural enemies per 25 tillers was subject to square root transformation.

There was no correlation between *R. padi* and maximum temperature (*R^2^* = 0.053, *P* = 0.329), minimum temperature (*R^2^* = 0.056, *P* = 0.290), rainfall (*R^2^* = 0.019, *P* = 0.222). There was a positive correlation between *R. padi* and rainfall duration (*R^2^* = 0.102, *P* = 0.048).


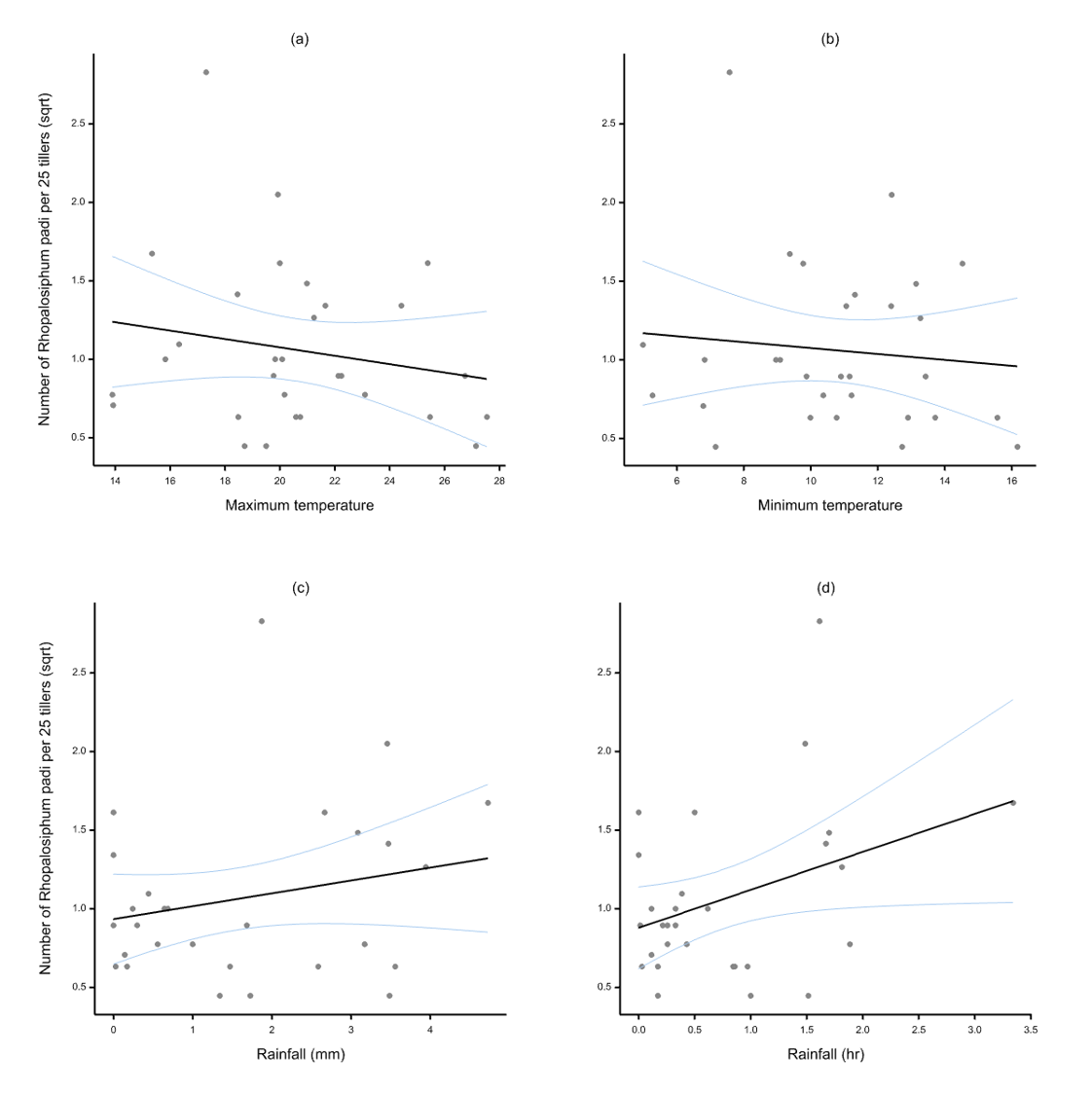


**Figure 2.** Correlation between number of *Rhopalosiphum padi* per 25 tillers and meteorological factors **a)** maximum temperature, **b)** minimum temperature, **c)** rainfall (mm), **d)** rainfall duration (hr). Number of natural enemies per 25 tillers was subject to square root transformation.

There was no correlation between *S. avenae* and maximum temperature (*R^2^* = 0.014, *P* = 0.465), minimum temperature (*R^2^* = 0.014, *P* = 0.807 rainfall (*R^2^* = 0.015, *P* = 0.238). There was a positive correlation between *S. avenae* and rainfall duration (*R^2^* = 0.116, *P* = 0.037).


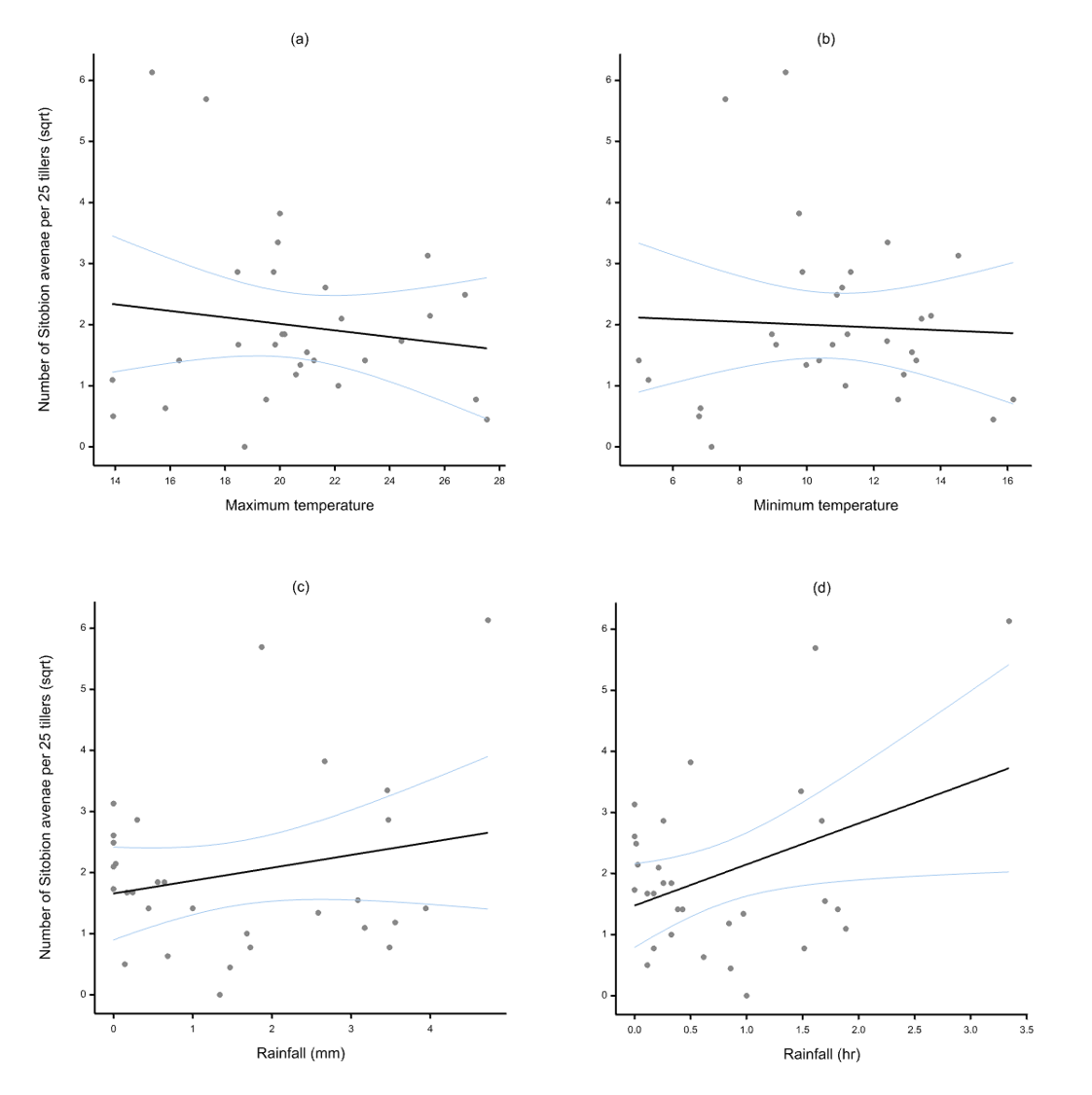


**Figure 3**. Correlation between number of *Sitobion avenae* per 25 tillers and meteorological factors **a)** maximum temperature, **b)** minimum temperature, **c)** rainfall (mm), **d)** rainfall duration (hr). Number of natural enemies per 25 tillers was subject to square root transformation.

There was no correlation between *M. dirhodum* and maximum temperature (*R^2^* = 0.091, *P* = 0.672), minimum temperature (*R^2^* = 0.093, *P* = 0.335 rainfall (*R^2^* = 0.091, *P* = 0.335). There was a positive correlation between *M. dirhodum* and rainfall duration (*R^2^* = 0.106, *P* = 0.044).


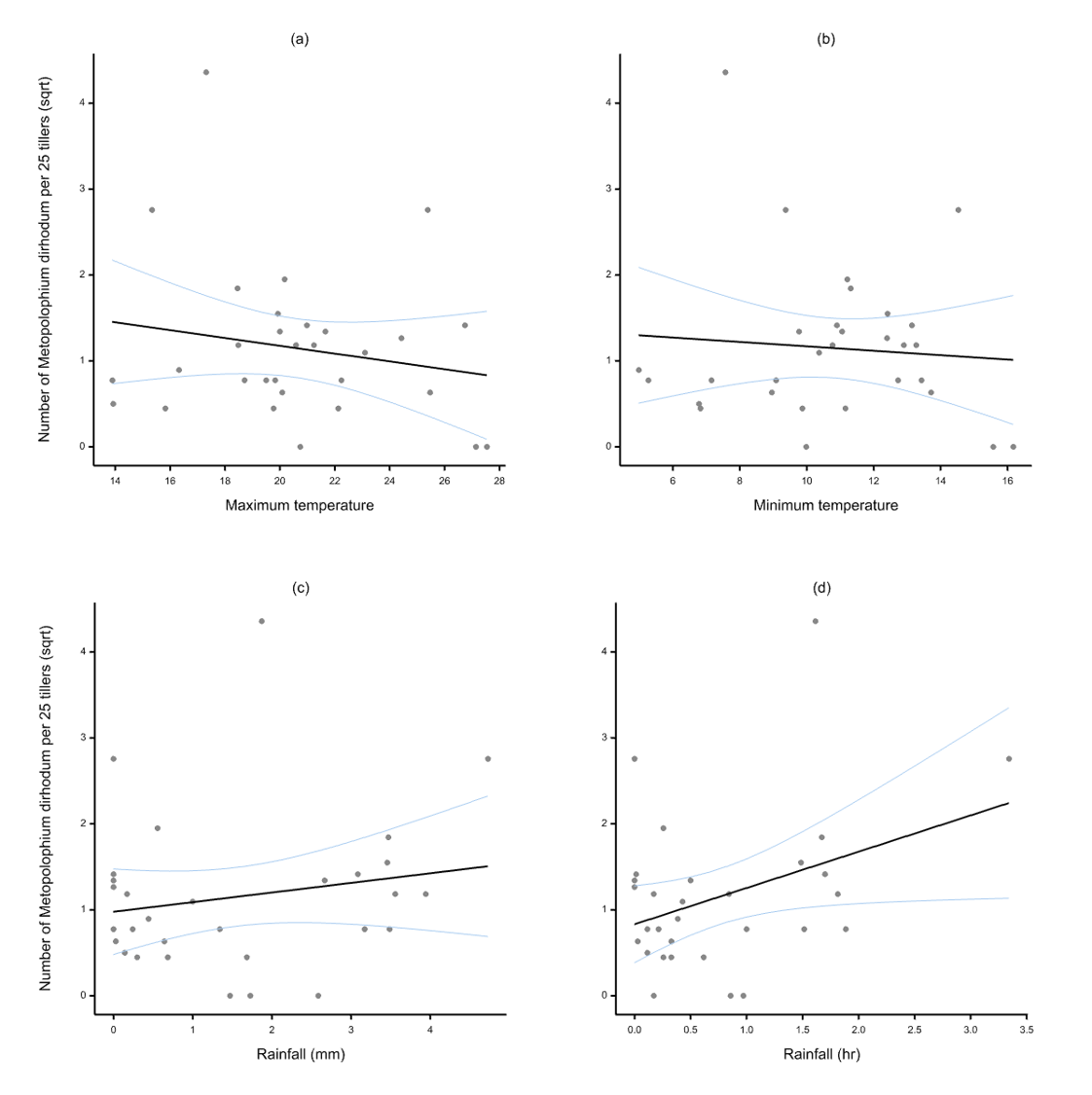


**Figure 4**. Correlation between number of *Metopolophium dirhodum* per 25 tillers and meteorological factors **a)** maximum temperature, **b)** minimum temperature, **c)** rainfall (mm), **d)** rainfall duration (hr). Number of natural enemies per 25 tillers was subject to square root transformation.

To determine whether correlations between meteorological factors maximum temperature, minimum temperature, rainfall and rainfall duration and natural enemies differ between the three insects, linear regressions were carried out on parasitised mummified aphids, lacewing eggs and larvae, and ladybird adults and larvae as well as overall natural enemy densities. Analysis was done using Genstat (2016, 19^th^ Edition, VSN International Ltd, Hemel Hempstead, UK).

There was no correlation between natural enemy population densities and maximum temperature (*R^2^*= 0.33, *P*= 0.169), rainfall (*R^2^*= 0.2, *P*= 0.314) or rainfall duration (*R^2^*= 0.8, *P*= 0.275), however, there was a positive correlation between natural enemy presence density and minimum temperature (*R^2^*= 0.101, *P*= 0.048) (Fig 5)


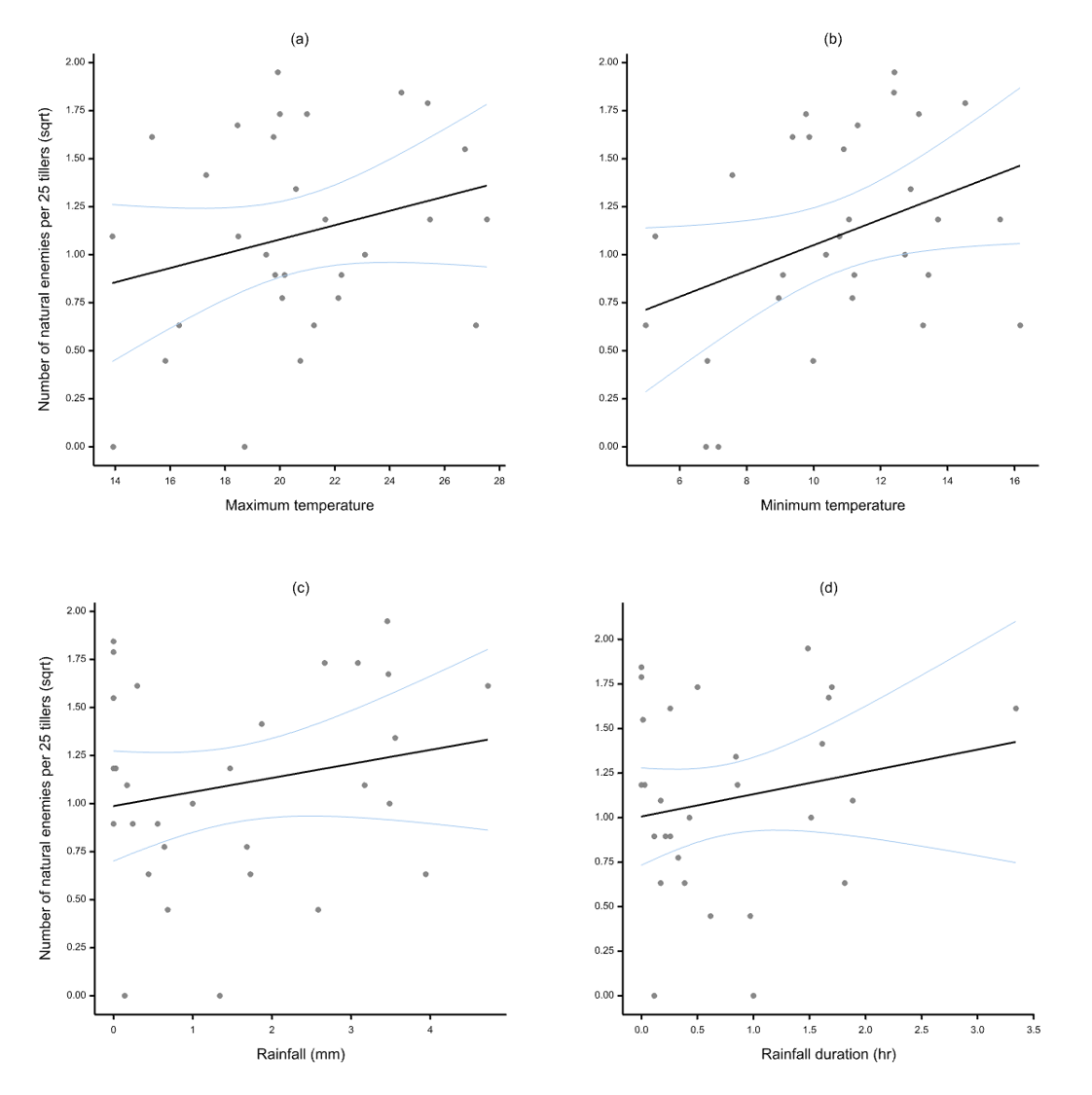


**Figure 5.** Correlation between number of natural enemies per 25 tillers and meteorological factors **a)** maximum temperature, **b)** minimum temperature, **c)** rainfall (mm), **d)** rainfall duration (hr). Number of natural enemies per 25 tillers was subject to square root transformation.

There was no correlation between parasitized mummified aphids and maximum temperature (*R^2^* = 0.031, *P* = 0.176), minimum temperature (*R^2^* = 0.066, *P* = 0.099), rainfall (*R^2^* = 0.054, *P* = 0.530) or rainfall duration (*R^2^* = 0.054, *P* = 0.707) (Fig 6).


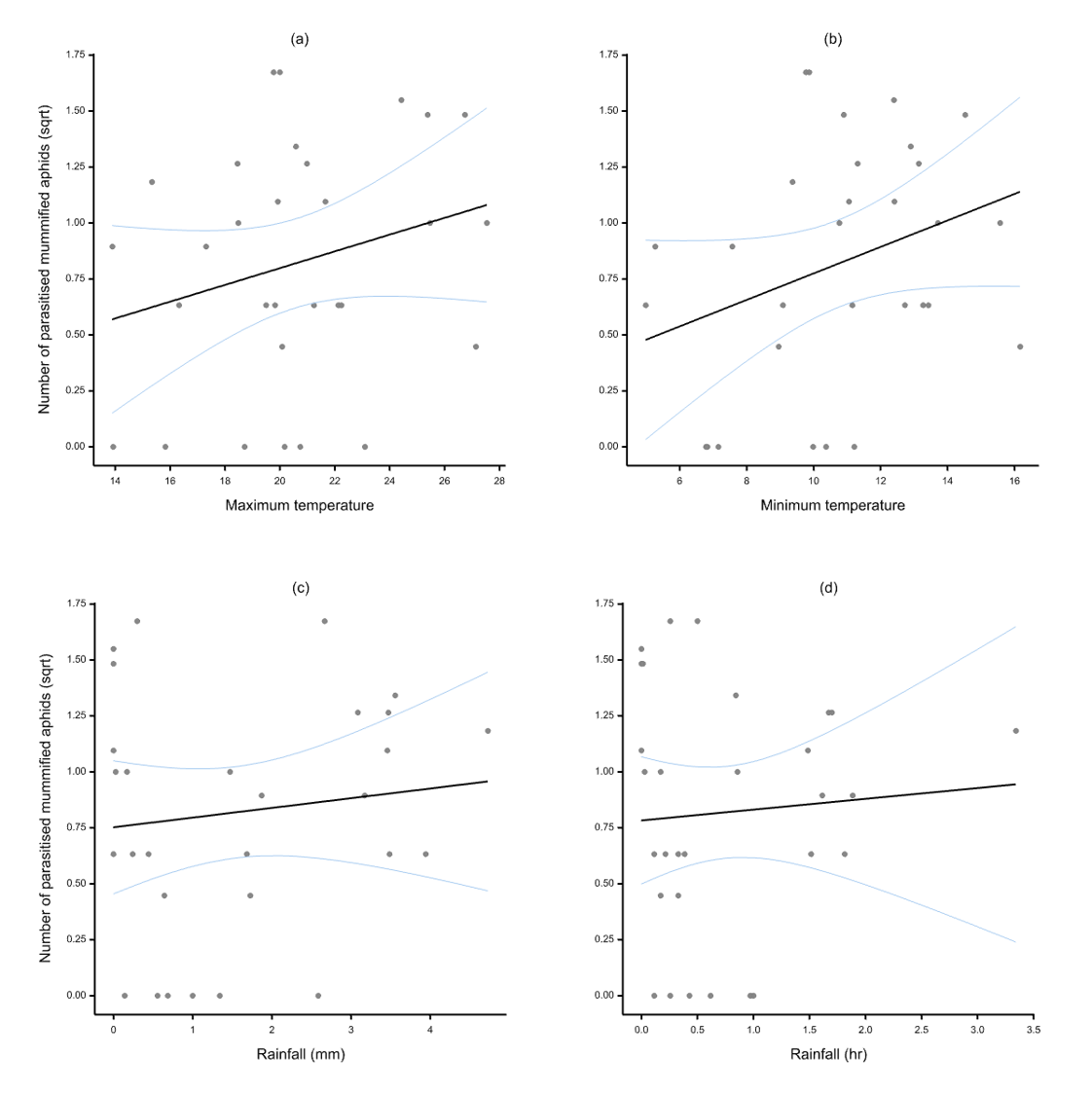


**Figure 6.** Correlation between number of parasitised mummified aphids per 25 tillers and meteorological factors **a)** maximum temperature, **b)** minimum temperature, **c)** rainfall (mm), **d)** rainfall duration (hr). Number of natural enemies per 25 tillers was subject to square root transformation.

There was no correlation between lacewing eggs and larvae, and maximum temperature (*R^2^* = 0.027, *P* = 0.192), minimum temperature (*R^2^* = 0.028, *P* = 0.187), rainfall (*R^2^* = 0.043, *P* = 0.331) or rainfall duration (*R^2^* = 0.043, *P* = 0.433) (Fig 7).


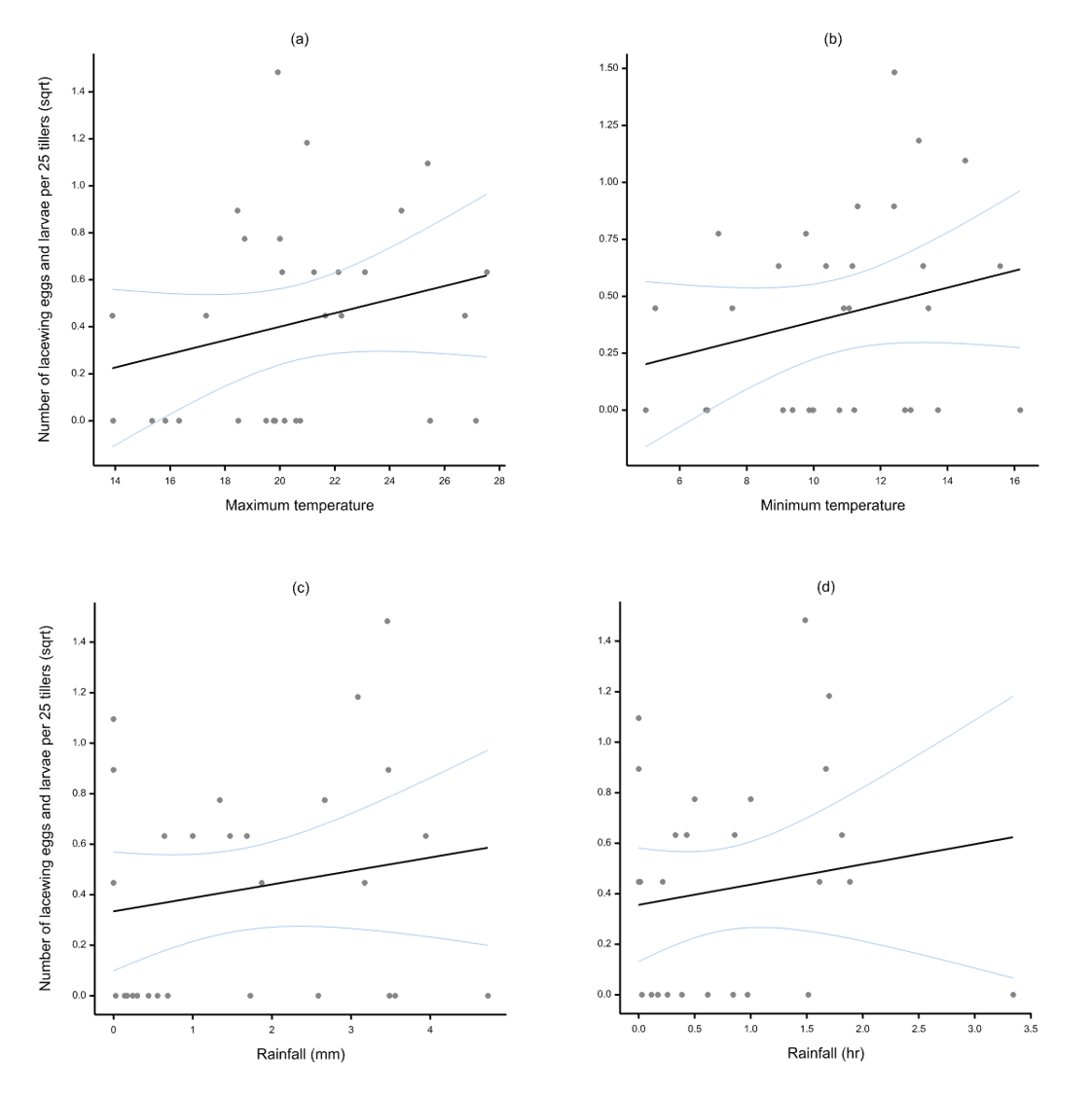


**Figure 7.** Correlation between number of ladybird adults and larvae per 25 tillers and meteorological factors **a)** maximum temperature, **b)** minimum temperature, **c)** rainfall (mm), **d)** rainfall duration (hr). Number of natural enemies per 25 tillers was subject to square root transformation.

There was no correlation between ladybird adults and larvae, and maximum temperature (*R^2^* = 0.030, *P* = 0.370), minimum temperature (*R^2^* = 0.031, *P* = 0.588), rainfall (*R^2^* = 0.029, *P* = 0.182) or rainfall duration (*R^2^* = 0.055, *P* = 0.111) (Fig 8).

**
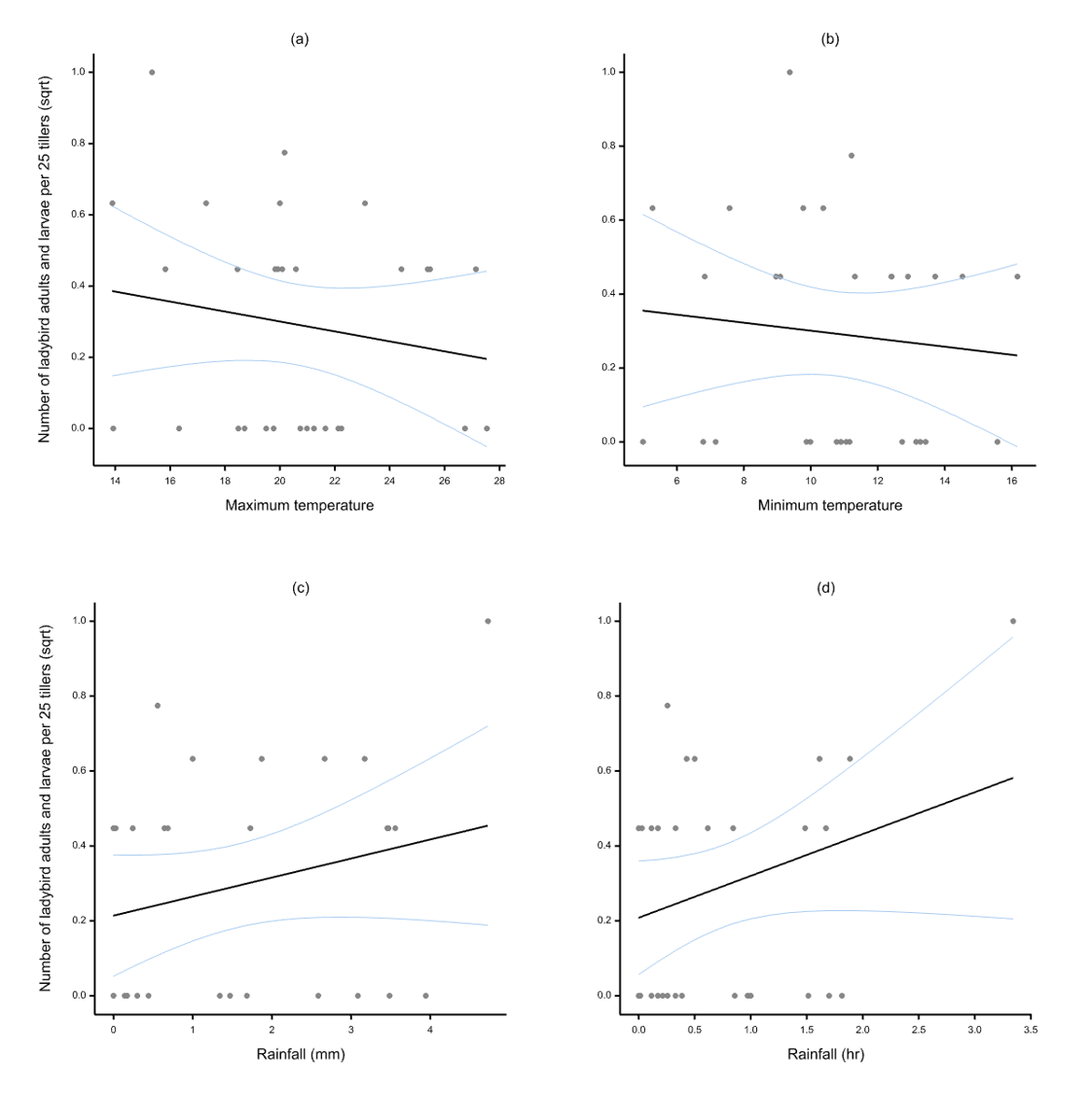
**

**Figure 8.** Correlation between number of lacewing eggs and larvae per 25 tillers and meteorological factors **a)** maximum temperature, **b)** minimum temperature, **c)** rainfall (mm), **d)** rainfall duration (hr). Number of natural enemies per 25 tillers was subject to square root transformation.
